# KdmB, a Jumonji Histone H3 Demethylase, Regulates Genome-Wide H3K4 Trimethylation and Is Required for Normal Induction of Secondary Metabolism in *Aspergillus nidulans*

**DOI:** 10.1371/journal.pgen.1006222

**Published:** 2016-08-22

**Authors:** Agnieszka Gacek-Matthews, Harald Berger, Takahiko Sasaki, Kathrin Wittstein, Clemens Gruber, Zachary A. Lewis, Joseph Strauss

**Affiliations:** 1 Division of Microbial Genetics and Pathogen Interactions, Department of Applied Genetics and Cell Biology, University of Natural Resources and Life Sciences, University and Research Center Campus Tulln, Tulln, Austria; 2 Health and Environment Department – Bioresources, Austrian Institute of Technology, University and Research Center Campus Tulln, Tulln, Austria; 3 Department of Microbiology, University of Georgia, Athens, Georgia, United States of America; 4 Helmholtz Centre for Infection Research GmbH, Department Microbial Drugs, Braunschweig, Germany; 5 Department of Chemistry, University of Natural Resources and Life Sciences, Vienna, Austria; Christian Albrechts Universitat zu Kiel, GERMANY

## Abstract

Histone posttranslational modifications (HPTMs) are involved in chromatin-based regulation of fungal secondary metabolite biosynthesis (SMB) in which the corresponding genes—usually physically linked in co-regulated clusters—are silenced under optimal physiological conditions (nutrient-rich) but are activated when nutrients are limiting. The exact molecular mechanisms by which HPTMs influence silencing and activation, however, are still to be better understood. Here we show by a combined approach of quantitative mass spectrometry (LC-MS/MS), genome-wide chromatin immunoprecipitation (ChIP-seq) and transcriptional network analysis (RNA-seq) that the core regions of silent *A*. *nidulans* SM clusters generally carry low levels of all tested chromatin modifications and that heterochromatic marks flank most of these SM clusters. During secondary metabolism, histone marks typically associated with transcriptional activity such as H3 trimethylated at lysine-4 (H3K4me3) are established in some, but not all gene clusters even upon full activation. KdmB, a Jarid1-family histone H3 lysine demethylase predicted to comprise a BRIGHT domain, a zinc-finger and two PHD domains in addition to the catalytic Jumonji domain, targets and demethylates H3K4me3 *in vivo* and mediates transcriptional downregulation. Deletion of *kdmB* leads to increased transcription of about ~1750 genes across nutrient-rich (primary metabolism) and nutrient-limiting (secondary metabolism) conditions. Unexpectedly, an equally high number of genes exhibited reduced expression in the *kdmB* deletion strain and notably, this group was significantly enriched for genes with known or predicted functions in secondary metabolite biosynthesis. Taken together, this study extends our general knowledge about multi-domain KDM5 histone demethylases and provides new details on the chromatin-level regulation of fungal secondary metabolite production.

## Author Summary

In this work we monitored by proteomic analysis and ChIP-seq the genome-wide distribution of several key modifications on histone H3 in the model fungus *Aspergillus nidulans* cultivated either under optimal physiological conditions (active growth) or less favourable conditions which are known to promote the production of secondary metabolites (SM). When we correlated the chromatin status to transcriptional activities in actively growing cells we found that the silenced SM gene clusters are flanked by heterochromatic domains presumably contributing to silencing but that the bodies of the clusters only carry background levels of any of the investigated marks. In nutrient-depleted conditions, activating marks were invading some, but by far not all transcribed clusters, leaving open the question how activation of these regions occurs at the chromatin level. Surprisingly, a large number of these gene clusters actually depend on KdmB for normal activation and it will be interesting to see in future how this protein thought to mainly act as repressor by removing positive H3K4m3 marks switches gears to activate transcription directly or indirectly.

## Introduction

Chromatin is the natural substrate for all eukaryotic nuclear processes such as transcription, replication, recombination or DNA repair. Chromatin structure is necessarily dynamic and the underlying mechanisms involve remodeling of nucleosomes as well as depositing and removing posttranslational modifications on N-terminal and central residues of histones proteins (HPTMs) present in the nucleosome octamer [1–4]. Some of these histone marks, such as acetyl groups on lysines, profoundly influence the chromatin landscape by neutralizing the positive charge of histones thereby weakening the interaction between nucleosomes and DNA and increasing chromatin accessibility [5]. HPTMs also work indirectly by providing binding sites for chromatin-associated proteins that promote or inhibit specific genomic functions. Notably, many HPTMs recruit additional chromatin-modifying enzymes that add new or remove existing marks, enabling cells to dynamically regulate chromatin structure in response to environmental or developmental cues. Fungi have served as model systems for chromatin studies and in many basic mechanisms they are similar to higher eukaryotes but in some aspects they are quite different and this fact allows evolutionary insights into the development of chromatin regulatory systems (reviewed in [6–8]). For example, there is ground-laying work from the filamentous ascomycete *Neurospora crassa*, where the molecular machinery relating heterochromatin formation and DNA methylation was deciphered [9–12]. Similar to animals also in *N*. *crassa* Heterochromatin Protein 1 (HP1), docks on di- or trimethylated lysine-9 on histone H3 (H3K9me2/3) to promote heterochromatin formation [13, 14] and in addition is important to maintain H3K27me3, another repressive mark, at facultative heterochromatin [15, 16]. This mark was found to span 6.8% of the fungal genome [17] corresponding to over 700 transcriptionally repressed genes, some of which are upregulated upon deletion of the H3K27 methyltransferase [16, 17]. While H3K27 methylation and elements of Polycomb Repressive Complex 2 (PRC2) responsible for depositing this mark are present in *Neurospora* and the *Fusarium* group of fungal pathogens (see below) this silencing mechanism has not been detected in *Aspergillus* species [18]. In addition, DNA methylation has not been found in the Aspergilli although a cytosine methyltransferase is functionally expressed in *A*. *nidulans* and has a role in regulating sexual development [19].

Mycotoxins, antibiotics, pigments and other low molecular weight natural products are summarized under the term of secondary metabolites (SMs). The *Fusarium* and *Aspergillus* genera are large groups of fungi comprising important plant and animal pathogens and they all produce (SMs) at certain developmental stages or under conditions of growth restriction, nutrient limitation and environmental stress (reviewed in [20–23]). It was shown initially in *Aspergillus nidulans* by genetic analysis that expression of the corresponding SMs biosynthetic genes, which are usually organized in gene clusters, is under chromatin control (reviewed in [24]). Under conditions of active growth SMs genes are silenced by H3 deacetylation [25, 26] as well as by the H3K9 methylation machinery of ClrD (KMT1/ DIM-5 homolog) and the hpo homolog HepA [27]. Interestingly, H3K4 methylation and a subunit of the COMPASS complex which are usually known to be associated with gene activation, also contribute to silencing although this has only been observed for a small subset of SM genes [28]. Several recent studies in a number of other fungi have implicated heterochromatin as a regulator of secondary metabolism and the production of virulence factors. In the plant pathogens *F*. *graminearum* (wheat and maize pathogen) and *F*. *fujikuroi* (rice pathogen) as well as in the fungal endophyte *Epichloë festucae*, H3K9me3 and H3K27me3 regulate expression of specific gene clusters responsible for the production of secondary metabolites [20, 23, 29–32]. H3K9me3 and HP1 were also shown to negatively regulate other virulence factors such as genes encoding small secreted proteins (SSPs) in *Leptosphaeria maculans* [29].

How HPTM patterns change as SM clusters switch from a repressed state to an active state is not completely understood. The requirement of histone H3 and H4 acetylation for SM gene expression is well documented in Aspergillus species through HDAC inhibitor studies and SAGA- complex mutants [33–35]. Interestingly, co-cultivation of *A*. *nidulans* cells with *Streptomyces rapamycinicus* led to an anomalous activation of several SM genes in the fungus [36] and this process is correlated with increased H3 acetylation of the corresponding genes and strictly dependent on GcnE, the catalytic subunit of the *A*. *nidulans* SAGA acetylation complex [37]. Also in *F*. *fujikuroi*, activation of the GA, bikaverin and fumonisin clusters was correlated with increased acetylation of H3K9 [38].

In contrast to acetylation, the role of histone methylation in fungal SM gene expression is much less clear. In *F*. *graminearum*, silent SM clusters are highly enriched for repressive H3K27me3, whereas trimethylated H3 lysine 4 (H3K4me3), an activating mark, is apparently excluded. Upon deletion of the H3K27 methyltransferase *kmt6*, the silent fusarin C and carotenoid clusters are activated, but H3K4me3 does not accumulate in these clusters [30]. A similar situation was shown in *F*. *fujikuroi* where increases in H3K4me2 were only observed in two genes of the gibberellin (GA) cluster. Similar to the case for H3K4me, expression of SM cluster genes in *F*. *graminearum* was not associated with increased H3K36me3 [30]. In contrast, H3K36me3 was gained for the sterigmatocystin (ST) and several other SM gene clusters in *A*. *nidulans* during activation [18, 39, 40].

H3K4me3 is an HPTM with important roles in transcription and this mark is generated by the COMPASS (Complex associated with Set1) protein complex containing the Set1 methyltransferase catalytic subunit in addition to several regulatory and scaffold proteins [41]. COMPASS is not essential in *A*. *nidulans* although synthetic lethality of Set1 and Swd1 subunits was found with mutations in mitotic regulators [42]. Generally, H3K4me3 has been shown to be recognized by three different domains associated with proteins of various functions. One recognition module is the PHD domain, present for example in the “Inhibitor of Growth” (ING) protein, which recruits histone acetyltransferase (HAT) and deacetylase (HDAC) complexes [43, 44]. H3K4me3 is also recognized by the double TUDOR domain of JMJD2A, a JmjC family demethylase that removes methyl groups from di- or trimethylated H3K9 [45] and by the tandem chromodomain of CHD1, an ATP- dependent nucleosomal remodeler [46] recently shown to be necessary for inhibition of intragenic initiation or initiation from cryptic promoters and thus maintaining normal transcript elongation [47]. Accordingly, H3K4me3 plays a central role in the chromatin regulatory network. Usually, H3K4me3 peaks at the transcription start sites (TSSs) and its occurrence is correlated with gene expression [48]. However, the Set1 protein also displays some moonlighting activities as it recruits deacetylase activity independently from the H3K4me3 mark and subsequently promotes heterochromatin formation and transcriptional repression at distinct loci in the fission yeast genome [49]. This evidently negative role of the COMPASS was also documented for regulation of SMs production in three different Aspergillus species carrying genetically engineered COMPASS mutations [28, 50, 51]. Silencing specific SM gene clusters might be related to previously documented subtelomeric silencing functions of the COMPASS complex [41] and mechanistically similar to the recently identified heterochromatin-promoting role in fission yeast [49].

Dynamic demethylation of lysine residues adds additional complexity to the modulation of transcription by lysine methylation [3, 52]. Recently we showed that KdmA, a JMJD2/JHDM3 family H3K9/36me3 demethylase [53, 54] can, in equal measure, positively and negatively influence gene expression in *A*. *nidulans* [18]. Here, we characterize another member of the JmjC demethylase family, KdmB, which acts on H3K4me3 *in vivo*, thus is assigned to the Jarid group of enzymes. Jarid (JMJ–AT-rich interacting domain-containing protein) subfamily demethylases have been shown to target di- and trimethylated H3K4 and are therefore generally considered to be repressors of gene transcription, though they can also act as activators [55]. For example the function of mammalian RBP2 (retinoblastoma binding protein 2, alias JARID 1A or KDM5A according to the new nomenclature [56]) in transcription regulation is context dependent. RBP2 represses transcription via H3K4me3 demethylation and association with an HDAC complex, however when associated with retinoblastoma protein (pRb), it activates certain genes in the mammalian genome [57]. Similarly, the *D*. *melanogaster* ortholog LID can repress transcription via H3K4me3 demethylation, however when associated with the MYC transcription factor, its demethylase activity is inhibited and consequently the LID-MYC complex mediates gene activation [58, 59]. These examples demonstrate that Jarid demethylases can act directly on their target genes in a context dependent positive or negative manner.

In this work we studied the Jarid-type demethylase in *A*. *nidulans* by reverse genetics and performed genome-wide HTPM profiling by mass spectrometry of histones, by ChIP analysis of H3K4me3, H3K9me3, H3K36me3 and H3 acetylation on K9 and K14 (H3Ac) modifications in wild type and compared the results with the KdmB mutant. We recorded these HPTM changes in parallel with the transcriptome under optimal physiological conditions promoting active growth (primary metabolism) as well as under stationary-phase conditions that lead to SM production (secondary metabolism). Comparison of ChIP-seq profiles with RNA-seq of the same cultures allowed us to correlate transcriptional changes with changes in chromatin landscapes across different conditions and genetic backgrounds. Histone proteomic analysis in wild type and the KdmB histone H3K4 demethylase mutant provided direct evidence for H3K4me3 as the dominant substrate for KdmB and confirmed that *A*. *nidulans* does not feature H3K27me3, the canonical facultative heterochromatic mark in other eukaryotes and responsible for SM gene silencing in a number of other fungi.

## Results

### KdmB possesses *in vitro* histone H3 demethylase activity

**Fig 1:**
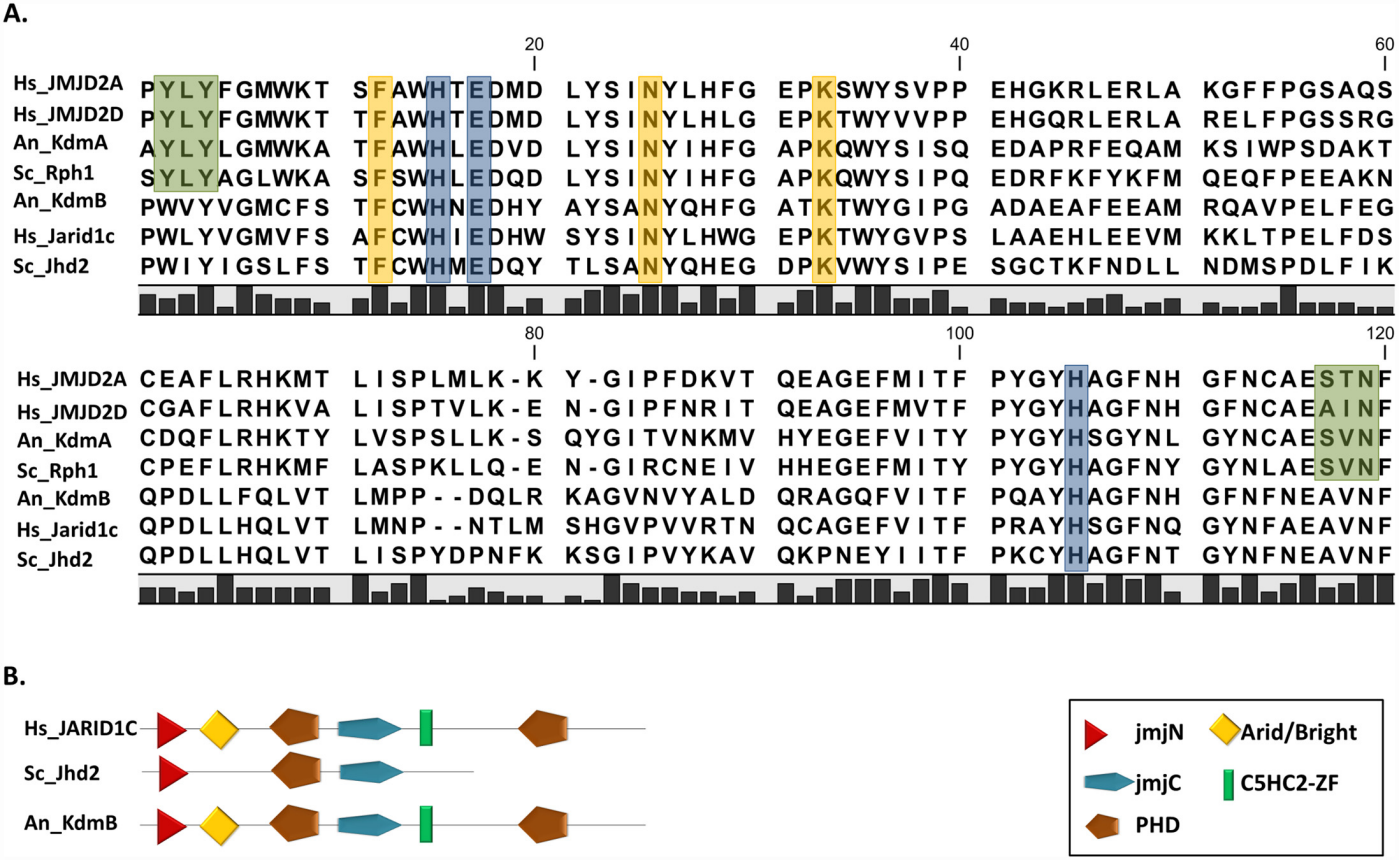
The catalytic domain of JMJD2 and JARID- JmjC family demethylases. **A**. Sequence alignment of JmjC domains of JMJD2 and JARID demethylases from human (Hs), *A*. *nidulans* (An) and *S*. *cerevisiae* (Sc). Conserved residues responsible for the catalytic activity are marked in yellow (α-ketoglutarate binding site) and blue (Fe^2+^ binding site). Specificity-determining residues for H3K9K36me2/3 are marked in green [61, 62]. **B**. Domain composition of JARID group H3K4me2/3 demethylases from human, *A*. *nidulans* and *S*. *cerevisiae*. KdmB possesses conserved histidine and glutamate as well as phenylalanine, asparagine and lysine residues responsible for Fe^2+^ ion chelating and α-ketoglutarate binding respectively, which are found in all catalytically active JmjC demethylases.

Based on the domain composition of the full length KdmB (AN8211) and detailed analysis of the amino acid sequences of the catalytic JmjC domains of histone demethylases from yeast to humans, KdmB was classified as a Jarid1-type histone H3 lysine 4 demethylase (Fig 1). Residues responsible for substrate recognition of Jarid demethylases are not known due to the lack of available crystallographic data, although the conserved amino acids required for substrate recognition in the JMJD2 subfamily of lysine K9 and K36 histone H3 demethylases (marked in green in Fig 1A) are not present in the Jarid group [60]. Domain analysis revealed that KdmB is more similar to the proteins from higher eukaryotes than from budding yeast. Specifically, we found that KdmB contains a putative ARID/Bright domain and a C5-HC2 zinc finger motif and an additional PHD domain at the C-terminus, which are both absent from the budding yeast homolog (Fig 1B).

To investigate the *in vitro* specificity of KdmB we heterologously expressed KdmB as a GST fusion protein in *E*. *coli*. KdmB has predicted molecular weight of 216 kDa but the resulting full size recombinant protein was not sufficiently soluble. Another construct producing a truncated KdmB protein without the second PHD domain, however, was readily soluble under native buffer conditions. This KdmB fusion containing residues 1 to 922 displayed an apparent mass of roughly 130 kDa (S1A Fig). *In vitro* demethylase assays (DeMt) were subsequently performed with purified GST-KdmB_(1–922)_ and calf thymus histones as a substrate. Products of the DeMt reactions were detected with modification-specific antibodies by Western blot (S1B Fig). Under our assay conditions, we found a decrease in trimethylation signals for all three tested lysine residues (H3K4me3, H3K9me3 and H3K36me3) and the strongest reduction in abundance was seen in H3K9me3. Acetylation was not reduced by the enzyme, as expected. Consistent with KdmB being a JmjC-type demethylase, the activity of the GST-KdmB_(1–922),_ fusion protein was dependent on the presence of the cofactors α- ketoglutarate and Fe^2+^ (S1B and S1C Fig). In our assay conditions we observed high standard deviations between independent replicates of H3K4me3 and H3K36me3-specific Westerns. This could be due to experimental variation in enzymatic activity of different batches of the purified recombinant enzyme.

The very broad substrate range of KdmB *in vitro* is unexpected for this Kdm5-family member because so far the identified and tested enzymes target either H3K4me2/3 (Jarid1 group enzymes) or H3K9/36me2/3 (Jmjd2 group). However, it is possible that the absence of PHD-finger 2, interacting proteins or the presence of the GST domain compromises substrate specificity in our assay. Although none of the KdmB orthologs identified so far demonstrated such broad substrate specificity *in vitro* [57, 63–66], the *in vitro* demethylase activity found in our assays suggests that this protein possesses histone demethylase activity.

### KdmB regulates histone methylation levels *in vivo*

**Fig 2:**
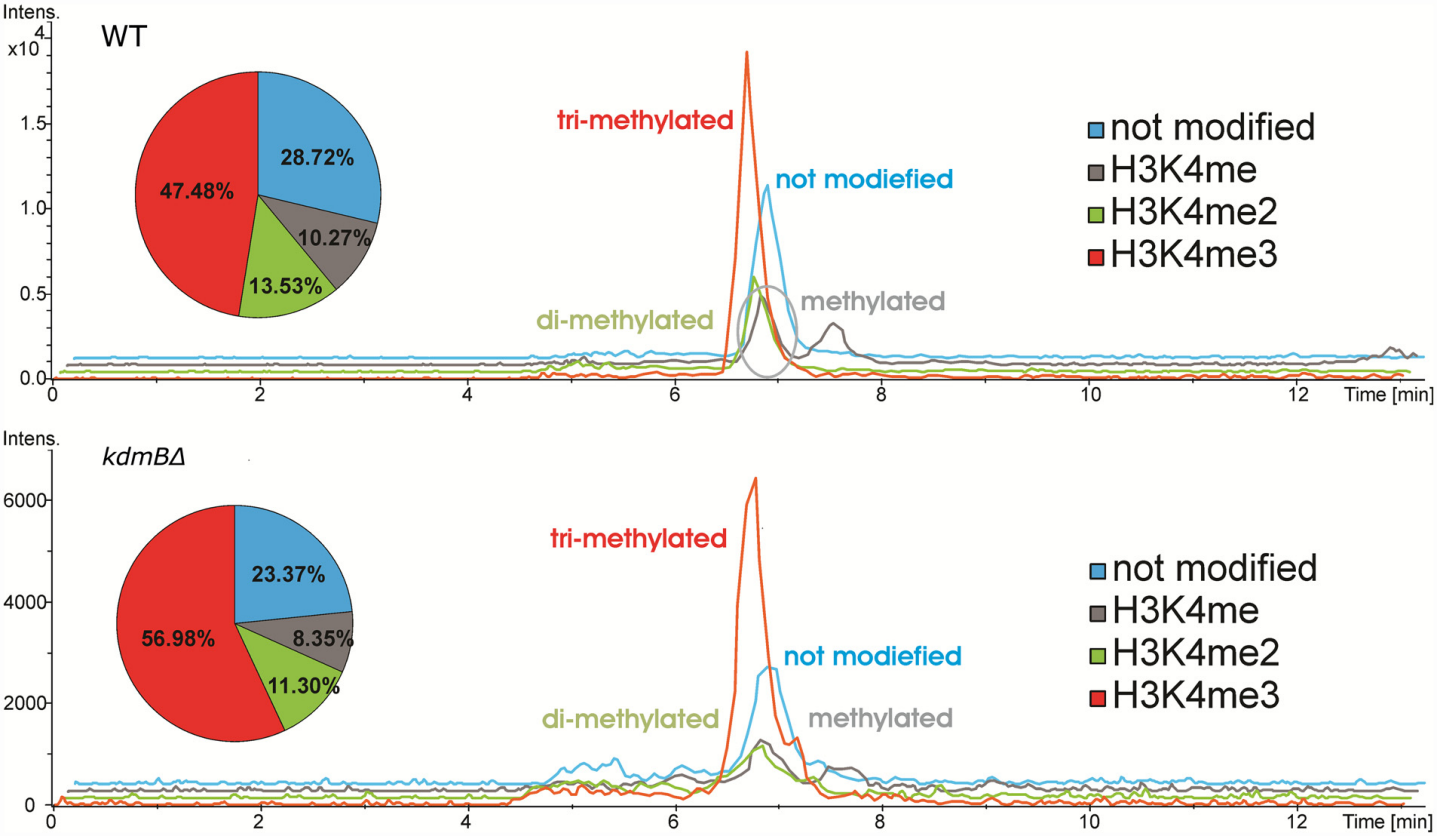
Levels of H3K4me3 are increased in *kdmBΔ* histones. MS/MS Base Peak Chromatograms (BPC) of tryptic histone digests analysing peptides T_3_-R_8_ of histone H3 show the different variants of methylated K4 and their ratios in the wild type (WT) and the *kdmBΔ* strain.

To determine whether KdmB can act as a histone demethylase *in vivo*, we performed LC- MS/MS on acidic extracted histones from actively growing *A*. *nidulans* wildtype and *kdmBΔ* cells (see Materials and Methods for description of gene deletion procedure). In wildtype, mass spectrometry revealed that 71.3% of H3K4 peptides contain at least one methyl-group at the K4 position. We detected all three forms of methyl-H3K4 peptides and found that H3K4me3 is the most abundant (47.5% of total H3K4 peptides), followed by di-methylated (13.5%) and mono-methylated H3K4 (10.3%) (Fig 2).

Notably, our measurements revealed an almost 20% increase in global H3K4 trimethylation in the *kdmBΔ* strain (57% H3K4me3). Because the levels of H3K4me2, H3K4me1 and unmodified H3K4 were concomitantly decreased in the mutant in roughly the same range as H3K4me3 increased we concluded that *in vivo* KdmB primarily acts to demethylate H3K4me3. The MS results also revealed that *in vivo* KdmB does not target H3K36me3 as these levels remained constant in histones of *kdmBΔ* cells (S2 Fig). Interestingly, the overall low marking of H3K9 by trimethylation (1.53% of the mapped peptides) was further reduced (to 0.2% of the mapped peptides) in the mutant. This contrasts the *in vitro* assay results which showed a strong H3K9me3 demethylating activity of recombinant KdmB (S1 Fig). The further reduction of H3K9me3 in *kdmBΔ* cells might be attributable, however, to an increase in the opposing, positively acting H3K4me3 mark limiting the possibility to deposit or maintain H3K9 trimethyl marks in the target regions. Strikingly, *in vivo*, global H3 N-terminal lysine acetylation (H3K9ac/K14ac) was increased almost by 20% in the *kdmBΔ* strain at the expense of unmodified peptides of H3 which are reduced from 22% in the wild type to 6% in the mutant (S2A Fig). This more abundant histone acetylation could be the consequence of both stronger marking by acetylases and/or reduced deacetylation. The latter mechanism has already been reported in connection with KdmB homologs in mammals where RBP2 (Jarid1a) and PLU1 (Jarid1b) recruit the Rpd3S histone deacetylase complex [57, 67]. Altogether, our data demonstrate H3K4me3 demethylation activity of KdmB in *A*. *nidulans* cells and lack of this activity in *kdmB* deletion cells leads to a shift in modification equilibrium with more abundant positive (H3K4me3, H3Ac) and less negative (H3K9me3) marks.

### H3K4me3 is localized to active genes in *A*. *nidulans*

To determine the genomic regions in which KdmB influences H3K4me3 levels we performed genome-wide ChIP analysis (ChIP-seq) in wild type and *kdmB*Δ strains with antibodies specific to H3K4me3 [28]. As our global histone analysis revealed a crosstalk of this modification to H3K9 trimethylation as well as to H3K9/K14 acetylation, we also included these marks in ChIP-seq. Although no changes occurred for H3K36 trimethylation at the level of bulk histones between WT and the *kdmB* mutant, we were interested if locus-specific differences occur and thus analyzed also this mark by ChIP-seq.

As previous studies from our lab and by others revealed a crucial function of chromatin structure and histone modifications on the regulation of secondary metabolite biosynthesis (SMB), we performed all subsequent RNA-seq and ChIP-seq experiments not only under the already described standard active growth conditions representing primary metabolism (PM; 17h liquid shake cultures, no nutrient limitation) but also under conditions *promoting secondary metabolism (48h liquid shake cultures*, *nutrient depletion*, *see* S3 Fig).

**Fig 3:**
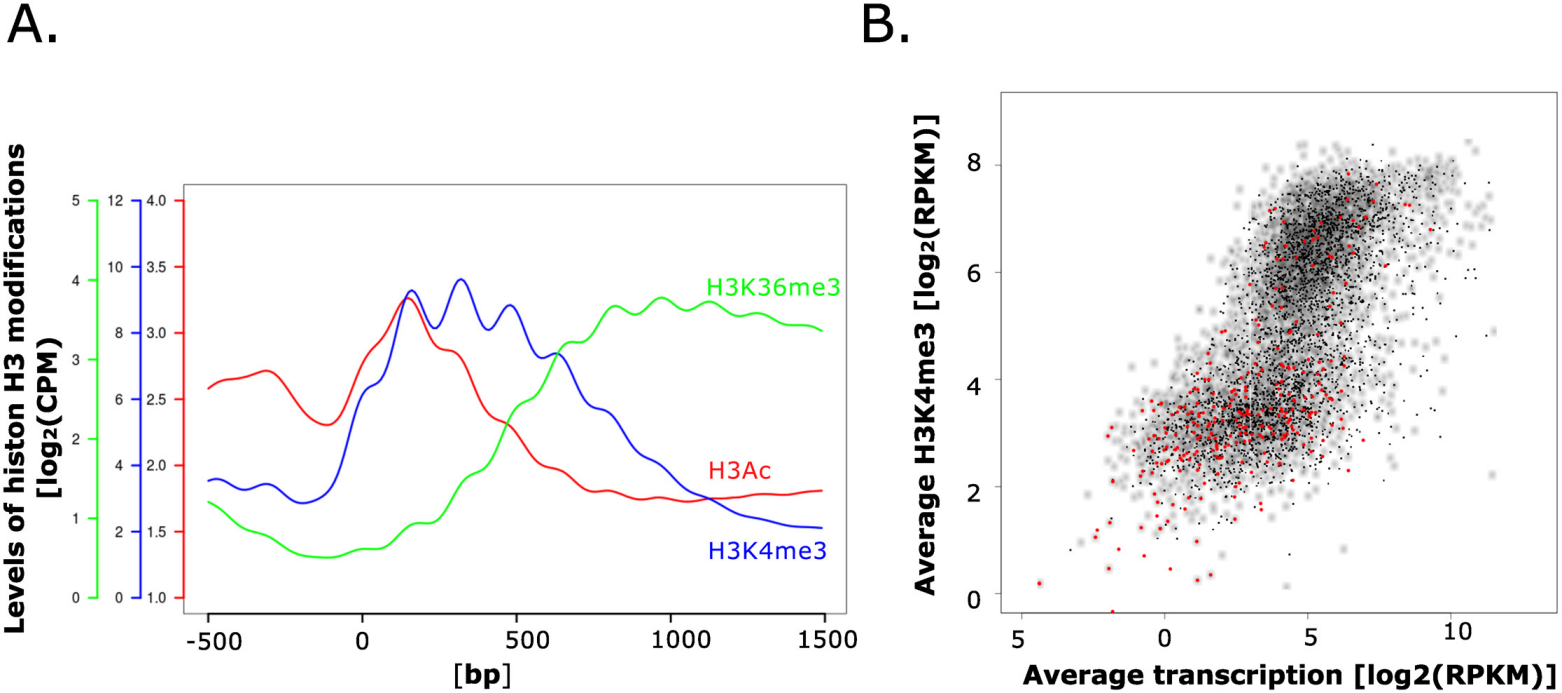
H3K4me3 localizes to actively transcribed genes. **A.** The metaplot depicts the enrichment pattern of H3K4 trimethylation (H3K4me3), H3K36 trimethylation (H3K36me3), and H3K9/K14 acetylation (H3Ac) for all genes on chromosome 4 in wild type actively growing cells (17h cultures). All genes are aligned to the predicted ATG (position 0) and analysed for a 2 kb window starting with 500 bp of their 5´UTR and promoter sequences (-500) followed by 1500 bp of their coding region (indicated positions within coding region 500, 1000 and 1500 bp). Counts were binned in 10-bp windows and averaged. **B.** The scatter plots shows the relationship between the average expression level and H3K4me3 enrichment. Values on the x-axis represent the average expression level of each gene determined by RNA-seq from both 17 and 48h cultures (see methods; log_2_ RPKM). On the y axis, normalized and H3K4me3 levels averaged over the whole gene (log_2_ RPKM) are shown for each of these genes. Grey dots represent constitutively expressed genes), and genes differentially expressed in the two tested conditions (with a p-value of p ≤0.001) are represented as black or red dots where the latter indicate differentially expressed genes involved in SM biosynthesis.

To monitor the distribution of the tested chromatin modifications along *A*. *nidulans* genes, we used chromosome IV as an example and plotted the wild type distribution of H3K4me3, H3K36me3, and H3K9/14ac across the promoters and open reading frames (ORFs) of all genes on this chromosome (Fig 3A). In this analysis, all genes are aligned to the predicted ATG (position 0) and read counts per million of mapped reads (CPM) are analysed in a 2 kb window starting with 500 bp of their 5´UTR and promoter sequences (-500) followed by 1500 bp of their coding region. This revealed that the pattern of modifications reflects the distribution observed in other model organisms including fungi [30, 68–70]. H3K4me3 was enriched in characteristic peaks spanning the first three nucleosomes (around 500 bp) of the coding region, whereas H3K36me3 was enriched near the 3’ regions of genes. Finally, H3 acetylation was enriched in the promoter, with highest levels apparent in the first nucleosome just downstream of the predicted translation start sites.

To explore the general relationship between H3K4me3 and transcription we quantified the average level of H3K4me3 in a 2 kb window around the predicted start codon of each gene (average CPM from -500 to +1500) and related this value to the average expression level (expressed as RPKM, reads per kilobase per million reads) of the corresponding gene in both culture conditions (PM and SM). In the resulting scatterplot (Fig 3B) two groups of genes became apparent, i.e. those that displayed high levels of H3K4me3 (log_2_ RPKM>5) and a second group that showed low to no H3K4 trimethylation (log_2_ RPKM≤5). Correlation of H3K4me3 levels with transcription of the corresponding gene revealed an overall positive correlation between H3K4me3 levels and transcript abundance (Fig 3B). This suggests that, similar to other well-studied models, H3K4 trimethylation is a marker for actively transcribed genes.

### *In vivo* KdmB is a H3K4me3 but not a H3K9me3 demethylase and influences transcription

**Fig 4:**
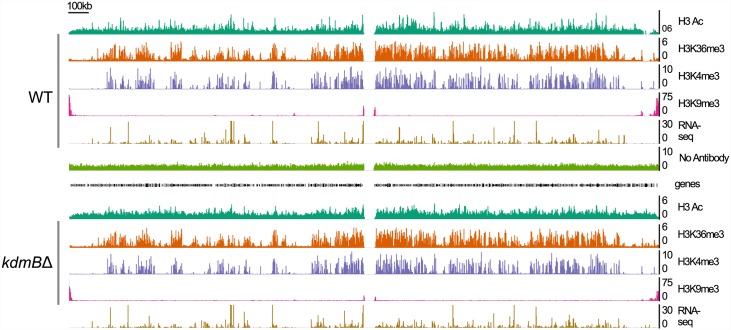
Genome viewer image presenting the chromatin landscape of chromosome IV in wild type (WT) and *kdmB* deletion (*kdmB*Δ) cells grown in liquid shake cultures for 17 hours (primary metabolism). RNA-seq represents transcriptional activity of the locus. A control ChIP (No Antibody) was performed to detect non-specific ChIP-seq signals.

To better characterize the function of KdmB in the context of transcriptional regulation we next compared by ChIP-seq the distributions of four histone modifications in wild type and *kdmBΔ* (Fig 4) under active growth conditions (PM) and during SM. The *kdmB* deletion did not cause any gross phenotypic changes in the mutant strain which was rather similar to the wild type in growth rates and nutrient consumption (S3 Fig). ChIP-seq combined with RNAseq analysis revealed the H3K4me3 enriched domains which coincide with transcriptional activity. In the example shown in Fig 4 we noticed, on the gross genomic scale, an overlap between the positively acting marks H3K4me3, H3K36me3 and H3Ac. In contrast, repressing H3K9me3 marks are enriched mainly in pericentromeric and subtelomeric regions and a few isolated H3K9me3 blocks exist (on the left arm of chromosome IV, for example).

**Fig 5:**
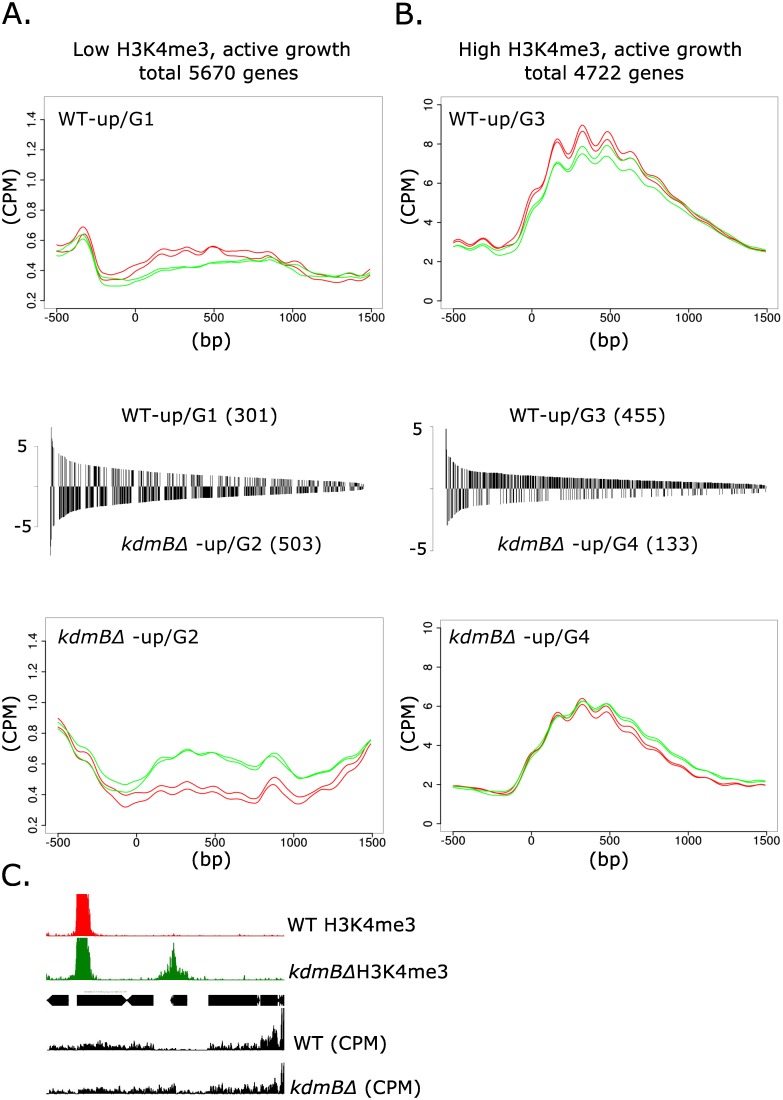
KdmB influences transcriptional activity and H3K4me3 levels. The Figure shows global correlation analysis of H3K4me3 levels and KdmB-dependent transcription in nutrient-rich culture cells (primary metabolism). All genes were categorized according to two criteria, i.e. at least 4-fold differential expression in WT and *kdmB*Δ as well as the degree of H3K4 trimethylation. This resulted in four categories, low (log_2_ RPKM ≤ 5, panel **A**) and high (log_2_ RPKM > 5, panel **B**) H3K4me3 levels and transcriptional up-/or downregulation in the *kdmB* mutant. For each open reading frame in these categories, the coverage in CPM (counts per million of reads) was calculated within a 2kb window around the predicted ATG (-500 to +1500) and represents the average enrichment level of this mark. Details on the bioinformatic procedure used to determine CPM values over all points in all genes are given in Materials and Methods. Red lines in the meta-plots indicate CPM values for the WT, while green lines indicate values obtained for *kdmB*Δ. The number of individual genes in each category and their level of de-regulation are shown in the bar-graph between the meta-plots. Each bar in the graph represents the differential expression value of an individual gene in this group. With this procedure four different correlation groups (G1 –G4) emerged, i.e. genes with low and high H3K4m3 levels requiring KdmB for normal transcription (WT-up/G1 and WT-up/G3, respectively) are expressed stronger in the wild type. Genes with low and high H3K4m3 levels under negative KdmB influence (*kdmB*Δ-up/G2 and *kdmB*Δ-up/G4, respectively) are stronger expressed in the *kdmB*Δ mutant. For all values, p<0.005 was set as threshold. **C.** Genome viewer image of one representative gene (locus AN6321) within the low H3K4me3 category in which *kdmB* deletion leads to gain of H3K4me3 (green boxed area) and higher transcription (*kdmB*Δ-up/G2 gene).

At the gross genomic scale the comparison of the chromatin landscape for H3K4me3 marks in chromosome IV between actively growing (17 h cultures) wild type and kdmBΔ cells did not reveal any obvious changes. Moreover, at this scale, no large domains were visibly changed for the other tested modifications (H3Ac, H3K36me3, H3K9me3). Because our mass spectrometry analyses uncovered increased H3K4me3 and H3Ac in the mutant, we reasoned that changes in the levels of these histone marks must occur at a subset of individual genes. To test this, we analyzed H3K4me3 levels in genes that were differentially expressed between wildtype and *kdmBΔ*. We first examined genes with low H3K4me3 levels [(log_2_ (RPKM)≤ 5] and found that 301 genes displayed higher expression levels in the wildtype (WT-up/Group 1, Fig 5A) suggesting that for this group KdmB is required for normal expression levels. In contrast, 501 genes had higher expression in *kdmBΔ* (*kdmB*Δ-up/ Group 2) which points to a repressing function of the protein in these loci. In the gene set featuring high H3K4me3 levels [(log_2_ (RPKM)> 5] we again identified both up- and down-regulated genes; 455 genes were expressed at higher levels in wild type (WT-up/Group 3) and 133 genes were expressed at higher levels in *kdmBΔ* (*kdmB*Δ-up/ Group 4).

The analysis showed that KdmB influences transcriptomes in both directions. For around 750 genes KdmB function is necessary for normal transcription, whereas for around 630 genes KdmB has a negative function. The repressive role of KdmB was found in both categories, i.e. on genes carrying low (*kdmBΔ*-up/G2) or high (*kdmBΔ-up/G* 4) H3K4me3 levels. Significantly, the group with normally low H3K4me3 (G2) displayed a marked increase in this histone mark in the *kdmBΔ* mutant concomitantly with increased transcript levels. One representative of this group is shown in Fig 5C for a gene (locus AN6321) which is basically not transcribed in the wild type but which gains both positive marks and transcripts in the *kdmB*Δ strain. Although we have not tested this directly, the strict correlation between increased H3K4me3 levels and transcription, along with the *in vitro* K4me3-demethylase activity of KdmB, suggests that at least some of these loci are direct targets of KdmB. A slightly different situation was found for the second gene set highly decorated with H3K4me3. Although a subset of these genes showed increased expression in the *kdmBΔ* mutant (*kdmBΔ-up/G* 4), this was not accompanied by an increase in H3K4me3 probably due to the already very high K4 methylation levels in the wild type. Consequently, a further increase would hardly be possible and thus the effect of *kdmB* deletion on H3K4 trimethylation is more subtle compared to genes generally not heavily marked by H3K4me3.

In contrast to the repressive function, KdmB also seems to have a positive role in transcription. *kdmB* deletion led to reduced expression of 750 genes belonging to both low (WT-up/G1) or high (WT-up/G3) H3K4me3 groups, accompanied by lower H3K4me3, on average, in the mutant. Based on these correlations we can conclude that KdmB function is required for normal expression of these roughly 750 genes, but whether KdmB directly targets these loci or indirectly affects transcription via the transcriptome network remains to be determined.

We also constructed metaplots of H3K4me3 distributions under SM conditions (S4 Fig). Under these growth conditions a similar correlation was observed, i.e. H3K4me3 levels were reduced in genes that were downregulated in *kdmBΔ*, whereas the genes upregulated in the mutant showed no drastic change (in the high H3K4me3 group) or somewhat higher H3K4 trimethylation. However, in locus-specific analysis by RNA-seq and ChIP-seq (see below), we also found some transcriptionally silent regions with high H3K4me3 as well as some highly transcribed genes with very low levels of this mark (see analysis below) indicating that specific genomic regions exist in which this general positive correlation between H3K4me3 and transcriptional activity does not apply.

### KdmB is specifically required for induction of SMB genes

**Fig 6:**
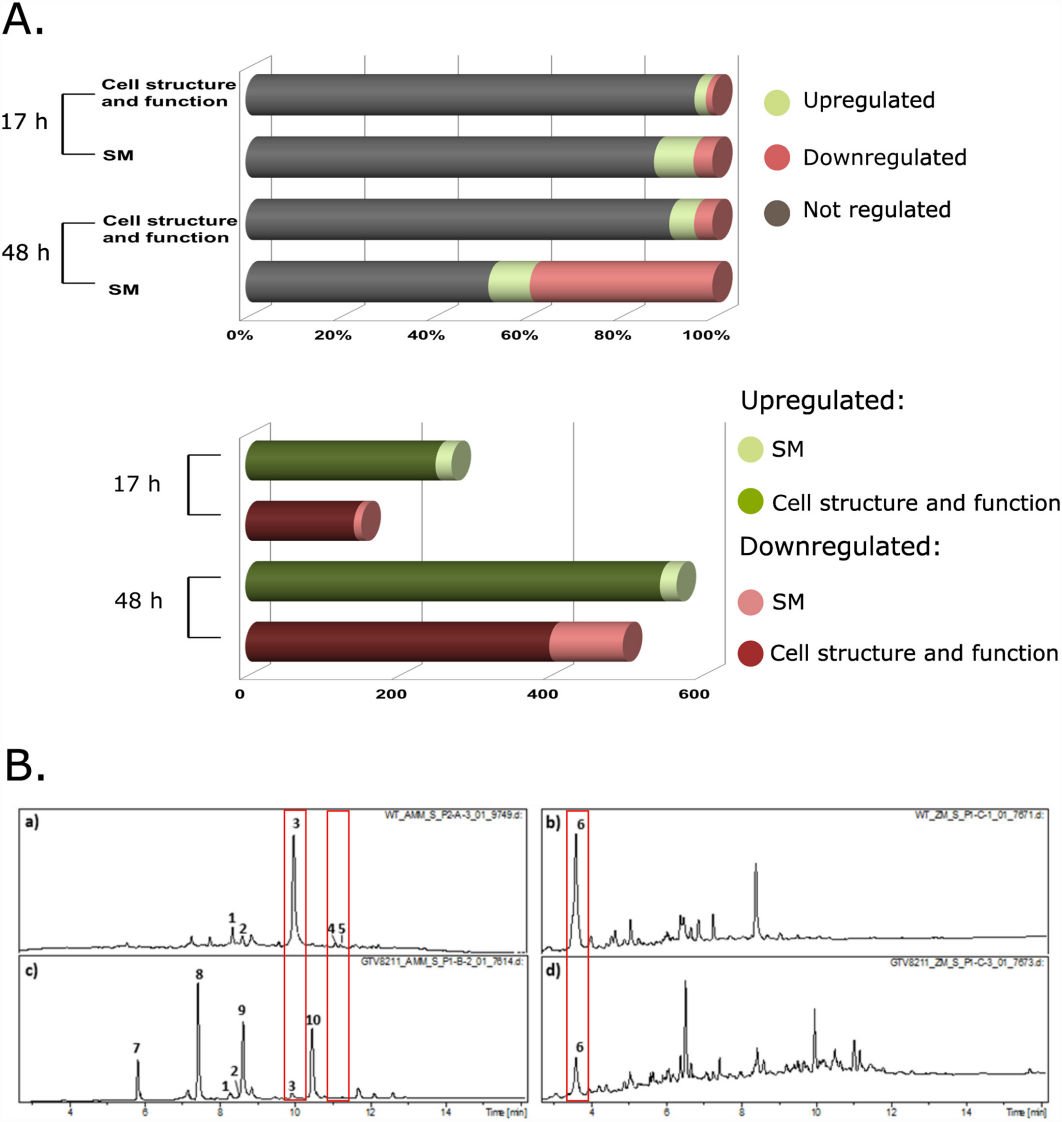
KdmB is required for normal induction of SMB genes. **A.** Upper panel: percentage of de-regulated genes in *kdmBΔ* during PM (17 h) and SM (48 h) with the division into SM cluster genes as annotated in [72] and basic metabolism genes (cell structure and function). Differential expression cut off was set to higher than 4-fold difference (log_2_ ≥ 2, p<0.05). The lower graph depicts the total number of de-regulated genes in *kdmBΔ* during PM (17 h) and SM (48 h) cultures,. **B.** HPLC-chromatograms of supernatant extracts from wild type and kdmBΔ cells growing for 48 hours in conventional AMM or in the special SM-promoting “ZM” medium. a) wild type extract (supernatant, AMM), b) wild type extract (supernatant, ZM), c) *kdmBΔ* extract (supernatant, AMM), d) *kdmBΔ* extract (supernatant, ZM). Peaks are assigned to compounds according to standards running in parallel analyses. (1) Austinol, (2) Dehydroaustinol, (3)-Sterigmatocystin, (4) Emericellamide C, (5) Emericellamide D, (6) Orsellinic acid, (7) 2,ω-Dihydroxyemodin, (8) ω-Hydroxyemodin, (9) 2-Hydroxyemodin, (10) Emodin.

Our initial correlation analysis of H3K4me3 and transcription revealed that among genes requiring KdmB for full transcription, the category of SMB genes was significantly enriched (p < 0.05). In further analysis, PM and SMB genes were separated based on functional categories and this bioinformatic approach created a large group of genes (5676 genes) predicted to be involved in general cellular functions and metabolism (category “cell structure and function” abbreviated CSF) and a smaller group of 149 genes predicted to be involved in SMB (category “SM clusters”). [71, 72]. Fig 6 shows that under PM conditions, approximately 5% of genes involved in CSF and 15% of genes assigned to SMB were affected by the *kdmB* deletion. The majority of *A*. *nidulans* SM cluster genes are not under/ during PM conditions, thus it is not surprising that differential expression of SM genes is largely restricted to the 48h cultures. Interestingly, several genes belonging to a gene cluster with a so far unidentified product were highly upregulated in the mutant at this 17h time point and this transcriptional pattern will certainly facilitate the future identification of the product derived from this predicted SM cluster.

In contrast to the mild effect on SM gene expression during PM conditions, KdmB-deficient cells showed significantly altered patterns of gene expression when cells were collected from cultures under SM conditions. Over 50% of all predicted SM genes were misregulated in the mutant. The majority of these displayed lower expression, while approximately 10% of SM genes showed higher expression in the *kdmBΔ* strain (Fig 6A, upper panel). In contrast, during the same culture condition only ~10% of genes not involved in SM were differentially transcribed in *kdmBΔ*. These data demonstrate that KdmB is required for normal induction of the majority of SM clusters in *A*. *nidulans*. It is probably relevant to note that the defect in SM cluster activation in the *kdmB* mutant is not due to a lack of wide-domain activator expression as *laeA*, *veA*, *velB* and *velC* are normally transcribed in the mutant (changes between WT and *kdmB*Δ log_2_ ≤ ± 1,7).

The lower panel of Fig 6A presents the number of deregulated genes within each category and time point. During primary metabolism (17h) KdmB function is required for a relatively small number of genes (143 genes in CSF and 10 genes in SM). In contrast, in the nutrient limited 48h cultures gene expression profiles are changed considerably in the mutant: 598 genes (401 CSF and 97 SMB genes) require KdmB function for normal expression and 569 genes (547 CSF and 22 SMB genes) are negatively influenced by the regulator. These data suggest that KdmB is primarily required during the stationary phase and obviously plays an important role for the expression of the majority (97 out of 149 of genes involved in SMB

We also tested whether transcriptional changes in *kdmB*Δ were correlated with changes in SMB biosynthesis. For this we performed HPLC-MS/MS analyses of cultures grown in two different media, i.e. in conventional minimal medium used throughout the studies (AMM) and in a specialized SM-promoting ZM medium (see Materials and Methods section). The comparison of WT and mutant culture extracts, grown in AMM medium, revealed a strongly decreased production of sterigmatocystin and emericellamides C and D (Fig 6B, left chromatograms) but other metabolites such as emodin and its derivatives were increased in *kdmBΔ* (Fig 6B, chromatograms a and c). However, our RNA-seq data showed that genes encoding for enzymes involved in emodin biosynthesis embedded in the *mdpL-A* monodictyphenon pathway are not differentially expressed between WT and the *kdmB* mutant (S13 Fig). To accommodate these differences, we speculate that the decreased transcription of other secondary metabolite clusters, such as the sterigmatocystin cluster, may lead to higher levels of available emodin precursors, such as acetyl-CoA and malonyl-CoA, and thereby to an increased synthesis of emodin derivatives. ZM culture extracts revealed reduced levels of orsellinic acid in *kdmBΔ* (Fig 6B, right chromatograms), consistent with our RNA-seq data showing a decreased expression from the orsellinic acid gene cluster in the *kdmB* deletion (S10 Fig). The complete list of identified metabolites together with LC-MS and LC-MS2 data are shown in the S3 Table.

### KdmB influences the H3 acetylation, but not the H3 K36 methylation landscape

We also carried out correlation analyses between H3 acetylation and H3K4 methylation in genes which are differentially regulated in the *kdmB* mutant (S5 Fig). For those genes where KdmB is required for full expression and which are consequently higher transcribed in the wild type (categories WT-up/G1 and G3) H3 acetylation levels are also higher, independently of H3K4 trimethylation. The same is true for genes which are negatively influenced by KdmB (*kdmB*Δ-up/G2) but only if H3K4me3 levels are low. On the contrary, genes with high H3K4me3 levels under negative KdmB influence (*kdmB*Δ-up/G4), acetylation levels are lower than in the wild type despite higher expression of the corresponding genes in this group. The molecular basis of this effect has not been investigated further in this study but it would certainly be interesting to determine if KdmB impacts acetylation indirectly or directly through protein interactions with HDACs or HATs.

We also examined a possible influence of KdmB on the distribution of H3K36me3 in genes expressed under primary metabolic conditions (S6 Fig). We have previously shown that this mark is associated with active transcription and that, at some tested loci, the trimethylated H3K36 state is removed by KdmA, another *A*. *nidulans* JmjC-containing protein belonging to the KDM4 family [18]. The vast majority of *A*. *nidulans* genes are highly decorated by this mark under PM conditions (around 9,100 genes). We did not observe significant differences in the levels or in the distribution of this mark in the *kdmB*Δ strain neither in this group nor in the group carrying low H3K36me3 levels (1249 genes. This indicates that KdmB is not a demethylase of trimethyl-H3K36 *in vivo*. Around 13% of the 9,100 genes are de-regulated in the *kdmB* mutant strain but despite this differential expression there are no significant differences in the associated H3K36 trimethylation levels. This means that, at least for the gene set in which KdmB influences transcription, it does not do this *via* manipulating H3K36me3 levels.

### Many SM clusters are located near H3K9me3 domains

The genome-wide distribution pattern of H3K9me3 supports the previously reported low levels of H3K9 trimethylation in *A*. *nidulans* wild type cells where we found approximately 1.5% of peptides carrying this mark. [18]. In ChIP-seq, the H3K9me3 pattern correlates with AT-rich domains flanking the subtelomeric regions but also includes sites along the chromosome arms, as shown on the left arm of chromosome IV (Fig 4). Inspection of H3K9me3-associated regions revealed that many SMB gene clusters such as the penicillin (S7 Fig), sterigmatocystin (S8 Fig), austinol (S9 Fig), orsellinic acid (S10 Fig) and terrequinone A (S11 Fig) are flanked by H3K9me3 domains at either one (e.g. the ST and TDI clusters) or at both sides (e.g. the PEN cluster) of the cluster. Whether these structures are functionally relevant for the regulation of SM gene clusters remains obscure but possible since deletion of the H3K9 methyltransferase gene *clrD* or of *hepA*, the gene coding for the protein recognizing H3K9me3, lead to up-regulation of genes within these clusters [27]. The observation that many H3K9me3 blocks are found in close proximity to SMB gene clusters raises the possibility that higher order chromatin structures or a as yet unstudied set of modifications may be important for normal regulation of SM gene expression, consistent with prior genetic analyses [27, 29, 31, 32].

However, we have also found several SM clusters such as the asperthecin (S12 Fig) and monodictyphenone (S13 Fig) cluster without such H3K9me3 borders. Interestingly, these clusters are not activated under the standard SM growth conditions used here (48 h cultures and nutrient deprivation). Instead, the MDP cluster is only expressed to detectable levels in a strain lacking the CclA regulatory subunit of the COMPASS complex which is responsible for H3K4 di- and tri methylation [28] and APT is highly expressed only in an *A*. *nidulans* mutant lacking SUMO, the small ubiquitin-related modifier protein known to profoundly regulate chromatin structure and function [73, 74]. Hence, absence of the H3K9me3 blocks might be correlated with special requirements for activation whereas SMB gene clusters activated under standard SMB conditions feature H3K9me3-flanking domains.

### SMB gene clusters have a characteristic chromatin signature

**Fig 7:**
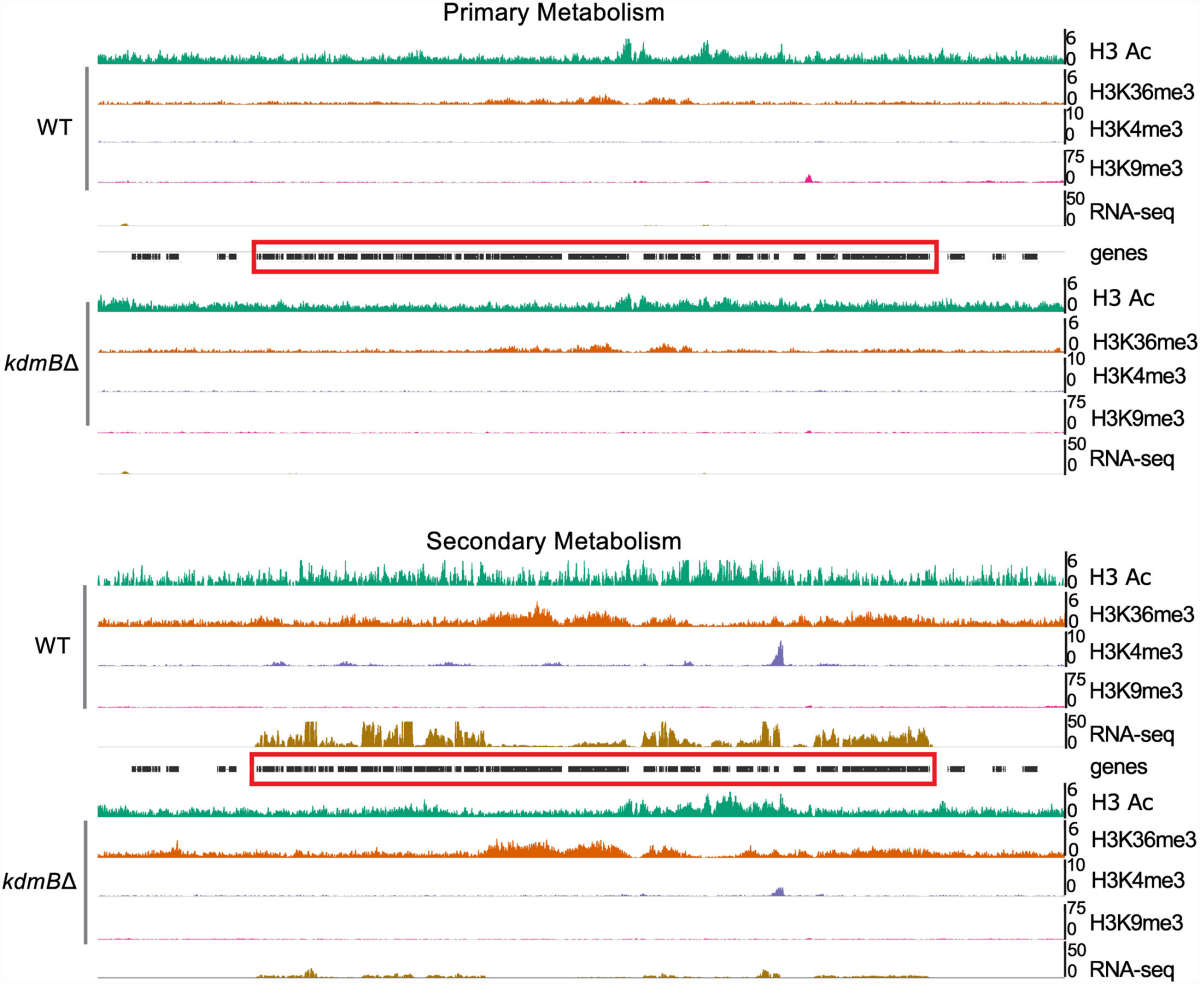
Chromatin landscape of the sterigmatocystin gene cluster. The ST cluster is indicated within the red box, the colour key is in the black box at the bottom of the figure. **A**. In both WT and *kdmBΔ* the ST cluster in 17 h cultures (PM) remains silent and lacks the investigated histone marks with the exception of H3K9me3 at *stcC*). **B.** The ST cluster is strongly induced at 48h (SM) in the wild type and the cluster genes gain low levels of H3K4me3, H3K36me3 and H3Ac. Levels of H3K9me3 at *stcC* are decreased in comparison to the flanking H3K9me3 domains under these SM conditions. In *kdmBΔ* the ST cluster remains almost silent. Unlike in the WT, besides low levels of H3K36me3, other activating histone marks are not detected at the ST locus in *kdmBΔ*.

Correlation of H3K4me3 with transcriptional activity suggested that SMB gene clusters carry low levels of this mark even when they are strongly transcribed (see Fig 3). Inspection of ChIP-seq data from these regions confirmed that H3K4me3 is underrepresented in such clusters, as shown in the example of the well-studied sterigmatocystin gene cluster (Fig 7).

When in conditions of primary metabolism, cluster genes are silent and are not associated with H3K4me3 but surprisingly, this mark is not established at most genes even when the cluster is fully activated (Fig 7). Eventually, a single strong H3K4me3 peak occurred around the 5´end of *stcD*, a gene coding for an unknown function but co-regulated with the sterigmatocystin biosynthesis cluster [75]. Qualitatively, the two other tested activating marks H3K9/K14 acetylation and H3K36 methylation seem to increase around 5´ and 3´ends of the ORFs, respectively, in the activated cluster. A very similar picture emerged from the analysis of other clusters (S9–S13 Figs) and in each case, as expected, no major differences in the H3K4me3 profiles became apparent between the *kdmB*Δ mutant and the wild type.

**Fig 8:**
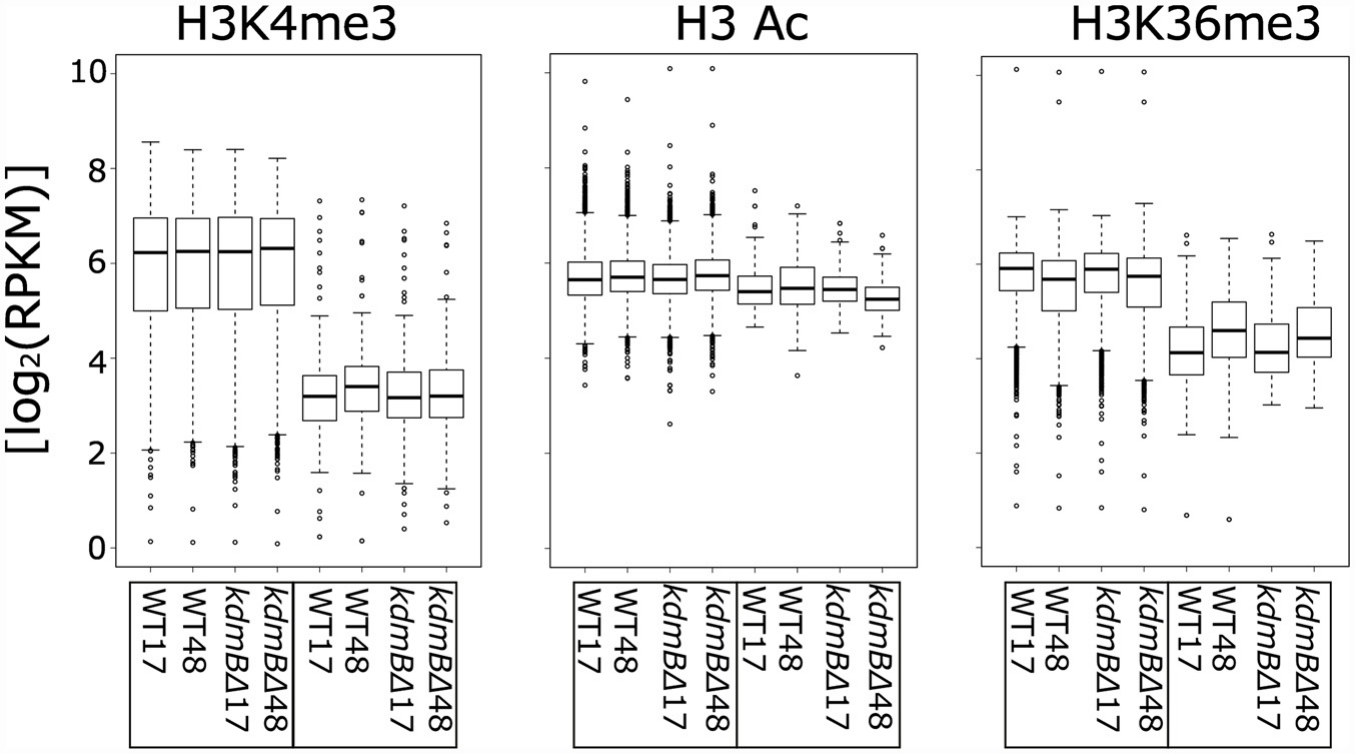
Boxplots of ChIP-seq results for Wild Type (WT) and *kdmBΔ* strains analysed for the two growth-phase dependent conditions, i.e. the two time points of harvesting after 17h (primary metabolism, PM) and 48h (secondary metabolism, SM). The [log_2_ (RPKM)] values at the two time points are given for the analysed chromatin modifications (H3K4me3, H3Ac or H3K36me3) and the gene set is divided into functional categories related to “Cell structure and function” (5676 genes) and to genes belonging to “SM clusters” (149 genes). H3K4me3 median of the log_2_ (RPKM) values for cell structure and function is higher than for SM clusters in all strains and conditions. The differences in H3 acetylation (H3Ac) and H3K36 trimethylation (H3K36me) are not significant between the categories and time points.

To test our qualitative impression for significance we performed statistical analysis of our ChIP-seq data for differences in H3K4me3, H3Ac and H3K36me3 marks in PM and SMB conditions in the wild type and in the *kdmB*Δ mutant. The bioinformatic separation into “Cell structure and function” and “SM clusters” categories applied for the transcriptome was also kept for ChIP-seq data analysis. The statistical analysis of ChIP data revealed a striking difference in H3K4me3 levels between the two categories. As seen in the box blot in Fig 8 genes involved in SM production are significantly less decorated by H3K4me3, regardless of the culture condition or the presence of KdmB.

Moreover, the pattern is not significantly changed in the *kdmB*Δ strain, suggesting that KdmB does not promote SM gene expression by directly regulating H3K4me3 within SM clusters. SM cluster activation leads to subtle increases in the level of H3K4me3, H3Ac or H3K36me3 associated with SM cluster genes, and this increase is not visible in *kdmBΔ* (Fig 5A). In summary, our ChIP-seq data revealed that *A*. *nidulans* SM clusters in comparison to genes involved in the cell structure and function have relatively low levels of activating histone marks, especially H3K4me3 and H3K36me3.

## Discussion

Di- and tri-methylation of histone H3K4 is associated with transcriptionally active chromatin. Removal of this modification is accomplished by members of the KDM5-family demethylases, typically resulting in repression of the targeted locus. In fact, the first characterized H3K4 demethylases LID2 [59] and RBP2 [57] were identified as transcriptional repressors. However, these proteins and all KDM5 members are composed of multiple domains which are necessary for the diverse functions these regulators play. For example, demethylase activity of KDM5 is only one of the important functions required for Drosophila development [76] [77]. In addition, some domains have been associated with gene activation, for example, mammalian Jarid1a is recruited to the *Per2* circadian gene promoter where it inhibits HDACs function and promotes transcription [78]. We also found in our study that deletion of KdmB has both activating and repressing effects. We found a genome-wide 20% increase in acetylated H3 N-termini and increased transcription of around 630 genes under standard growth conditions (nutrient sufficiency, primary metabolism) in strains lacking KdmB. These results provide evidence that the protein functions as a repressor that is able to remove H3K4me3 and perhaps recruit HDACs. Our data also demonstrate that KdmB is an H3K4me3 demethylase. The protein removed this modification *in vitro* and genes that are overexpressed in a KdmB-deficient mutant show increased H3K4me3. Unfortunately, from our data, we cannot deduce which part of this gene set is directly targeted by KdmB and which may be indirectly silenced through transcriptome network effects. ChIP analysis of KdmB tagged versions will be able to clarify this point in future.

On the other hand, for around 750 *A*. *nidulans* genes KdmB is required for full transcription. It is possible that KdmB mediates activation directly via one or more domains such as the potentially DNA-binding Zn-finger or ARID domains or the methylated histone binding PHD domain. However, deciphering how KdmB promotes transcription requires further investigation. Strikingly, the majority of the genes under positive KdmB control are related to the production of secondary metabolites. These small natural products are defense and signaling molecules of fungi produced during development, under stressful or nutrient-limiting conditions [23] and it is interesting that a general chromatin regulator such as KdmB takes up this specialized function in metabolism. We have shown that KdmB regulates (directly or indirectly) almost 5% of the genome during PM and over 10% during SM. The activation signal for the induction of genes involved in SMs production is transmitted *via* the so-called *velvet* activation complex containing also a protein termed LaeA that influences chromatin structure [27]. It will be interesting to determine if KdmB functions in this pathway. It is possible that KdmB could regulate SM gene expression by demethylation of SM regulatory proteins. Recently it was shown that several JmjC family demethylases can target non-histone substrates; however this function, to our best knowledge, has not been demonstrated for Jarid family demethylases [77, 79].

This role of KdmB in SMB gene activation appears to be independent of its histone demethylase enzymatic activity. In *kdmBΔ*, low levels of H3K4me3 and H3 lysine acetylation in SM gene clusters under activating conditions are likely the consequence of lower transcription at these *loci*.

One of the most striking features of silent *A*. *nidulans* SM clusters is a very low abundance or virtual absence of the four investigated histone marks within the borders of these gene clusters. At the moment we cannot exclude the possibility that other histone marks define the chromatin landscape within and around SM gene clusters. A large number of SM clusters, as exemplified here for sterigmatocystin (ST), penicillin (PEN), orsellinic acid (ORS), teraquinone (TDI), derivative of benzaldehyde 1 (DBA), austinol (AUS) and asperthecin (APT) are located in regions for which H3K4me3, H3K36m3 or H3Ac can hardly be detected. Even the monodictiphenone (MDP, S13 Fig) and asparthecin clusters (APT, S12 Fig), which are located within euchromatic regions, display a low-abundance of HPTMs. A distinguishing feature of these two clusters, which are not activated by the conventional SMB culture conditions applied here, is the lack of flanking by H3K9me3 domains which are characteristic for the majority of the analyzed SMB gene clusters (S7–S13 Figs). Although truncated KdmB demethylates H3K9me3 *in vitro* (S1 Fig) we did not see increased levels of this mark in the *kdmB*Δ mutant neither at specific loci nor at the genomic scale. This strongly suggests that *in vivo* H3K9me3 is not a target of KdmB. Moreover, no KDM5-type H3K9 demethylases have been described in other ascomycete fungi including *S*. *pombe* or *N*. *crassa*. In *A*. *nidulans* we even see genome-wide reduced levels of this mark in the mutant and it is likely that this reduced H3K9me3 is an indirect consequence of increased H3K4me3 or increased H3 acetylation. In addition to H3K9me3, the chromatin landscape changes slightly also for the other tested marks when the silent SM gene clusters are activated. The majority of the genes in these clusters gain H3K36me3 at their 3’ region and H3Ac at their 5’ region. Marking by H3K4me3, however, only occurs for a limited number of genes within these clusters such as some selected genes within the ST cluster (Fig 8) or the *orsD* gene positioned within the ORS cluster (S10 Fig).

A similar situation was recently reported in two different *Fusarium* species in which the H3K4 dimethylation level (H3K4me2) was compared to SMB gene transcription. In the rice pathogen *F*. *fujikuroi*, only two out of seven highly transcribed genes in the gibberellin cluster were significantly decorated with H3K4me2 [38] and also in *F*. *graminearum*, a pathogen of wheat and maize, genes in the fusarin C or the carotenoid biosynthesis clusters carried only background levels of this mark [30]. It is still remarkable that Liu and colleagues found an essential function of the H3K4 methyltransferase Set1 for the expression of the TRI gene cluster coding for deoxynivalenol biosynthesis [80] and in this latter study, H3K4me2 was clearly enriched over the background level and positively correlated with active transcription. Additionally, in contrast to our study, Connolly et al. [30] found H3K36me3 enrichment across the whole chromosome independent of transcriptional activity. These comparisons already highlight the high diversity of chromatin-based regulation in SMB gene expression within one single organism and even more between different organisms and which histone modifications are determining whether the SMB signal is transmitted to the transcriptional machinery or not. Surprisingly, the H3K4 demethylase KdmB plays an essential role in the activation process although this histone mark is not present in the targeted regions.

## Materials and Methods

### Strains, media, growth conditions and transformations

*A*. *nidulans* strains used in this study are listed in S1 Table. Experimental strains were obtained by transformation into an *nkuAΔ* strain, which reduces the frequency of non-homologous integration [81], or by sexual crosses. Genetic analysis was carried out using techniques as described by Todd et al. [82]. DNA transformation of *A*. *nidulans* was performed according to [83]. KdmB deletion cassettes were constructed using DJ PCR [84] with the *Aspergillus fumigatus riboB* gene as selectable marker, *riboB*+ transformants were recovered after transformation into *nkuA* strains. Southern analysis confirming the deletion of *kdmB* was performed as described elsewhere [18, 40]. AMM minimal media, complete medium, supplements and growth conditions were as described by Todd et al. [82]. ZM_1/2_ medium (molasses 0.5%, oatmeal 0.5%, sucrose 0.4%, mannite 0.4%, D-glucose 0.15%, CaCO_3_ 0.15%, edamine 0.05%, (NH_4_)_2_SO_4_ 0.05%) was used for promoting SM biosynthesis in the experiments analyzing metabolites by HPLC-MS/MS [85]. For LC-MS/MS, RNA-seq, ChIP-seq, DeMt assay, HPLC-MS/MS spores in concentration 4*10^6^/mL were inoculated into 200 mL liquid AMM and incubated at 180 rpm, 37°C for 17 h and 48 h. For SM cluster gene expression, ChIP and HPLC- MS/ MS analysis 10 mM sodium nitrate otherwise ammonium tartrate at 10 mM was added as nitrogen source.

### Protein expression and demethylation in vitro assay

*kdmB* cDNA was amplified using RevertAid Premium Reverse Transcriptase (Thermo Scientific, EP0732) and specific primers. Full length (1717 aa) and truncated versions (residues 1–922) cDNAs were cloned into pGEX-4T1 expression vector, sequenced, transformed and expressed in Rosetta cells. GST-KdmB (1–922) was purified using glutathione Sepharose 4B (GE Healthcare). Demethylation assay was performed as previously described [66]. Purified KdmB was incubated with calf thymus histones (Sigma, H9250) in demethylase reaction buffer (20mM Tris-HCl pH 7.2, 150 mM KCl, FeSO_4_ 20 μM, α-ketoglutarate 500 μM, ascorbic acid 500μM, ZnCl_2_ 1μM) for 3 to 10 h at 37°. Reaction was stopped by boiling for 5 minutes with 100 mM DTT Laemmli buffer; changes in lysine methylation were measured by Western blot with the specific antibodies (see ChIP section). The demethylation *in vitro* assay and Western blot were performed three times; negative controls were incubated without the cofactors for JmjC proteins (Fe^2+^ and α-ketoglutarate).

### Analysis of HPTM by western blot and LC-MS/MS

Mycelia from o/n liquid submerged cultures were harvested by filtration and frozen in liquid nitrogen. Histones were acid extracted as previously described [86], suspended in Laemmli’s SDS sample buffer and quantified with Pierce BCA Protein Assay (Thermo). 15 μg of purified histones, 1 μg of recombinant *Xenopus laevis* H3 as a negative control (Milipore, 14–441) and 2 μg calf thymus histones (Sigma, H9250) as a positive control were separated on 15% SDS-PAGE gel and subsequently transferred to nitrocellulose membrane (GE Healthcare) by electroblotting. Relevant histone modifications were detected with primary antibodies specific to H3K4me3 (Abcam, 8580), H3K9me3 (Active Motif, 39161), H3K36me3 (Abcam, 9050), histone H3 C-terminus (Abcam, 1791), H3Ac (pan-acetyl) (Millipore, 06–599) and anti- rabbit (Sigma, A0545) and anti- mouse (Sigma, A9044) HRP conjugated secondary antibodies. Chemiluminescence was detected with Clarity ECL Western Substrate and ChemiDoc XRS (Bio- Rad). Densitometric quantification of Western blot signals from demethylase reactions were performed with the ImagJ software. In total three independent blots of demethylase and the control reaction (without the cofactors) were quantified. Signal of respective HPTM were normalize to histone H3 C-term. Subsequently the signal of the control reaction was set to a value 1, consequently the presented results are the fold change to the control reaction. For MS analysis relevant histone H3 protein bands were cut out and digested in gel. The proteins were S-alkylated with iodoacetamide and digested with ArgC (Roche). The peptide mixture was analysed using a Dionex Ultimate 3000 system directly linked to a Q-TOF MS (Bruker maXis 4G ETD) equipped with the standard ESI source in the positive ion, DDA mode (= switching to MSMS mode for eluting peaks). MS-scans were recorded (range: 150–2200 Da) and the 6 highest peaks were selected for fragmentation. Instrument calibration was performed using ESI calibration mixture (Agilent). For separation of the peptides a Thermo BioBasic C18 separation column (5 μm particle size, 150*0.360 mm) was used. A gradient from 95% solvent A and 5% solvent B (Solvent A: 0.1% FA in water, 0.1% FA in ACCN) to 32% B in 45 min was applied, followed by a 15min gradient from 32% B to 75% B that facilitates elution of large peptides, at a flow rate of 6 μL/min.

### High throughput RNA sequencing (RNA-seq) and analysis

The fungal cultures were incubated in triplicates, RNA from each technical replicate was pooled and each experiment was performed twice to obtain two biologically independent sets with two technical replicates for each strain and condition. Illumina sequencing libraries were made from RNA samples according to TruSeq RNA Sample prep kit v2 (Illumina) following the manufacturers protocol with 1μg total RNA input. 50 bp single end sequencing was performed using a HiSeq Illumina sequencer. Obtained sequences were de-multiplexed, quality controlled and mapped on the *Aspergillus nidulans* genome assembly (A_nidulans_FGSC_A4_version_s10-m03-r07). Mapping was performed using Novoalign (NovoCraft) and reverse transcripts were counted using python script HTSeq [87]. Normalization and statistics were done using R/Bioconductor and the limma and edgeR packages, using mean-variance weighting (voom) and TMM normalisation [88]. A significance cut-off of p < 0.01 (adjusted for multiple testing by the false discovery rate method) was applied for analysis. R plots used the ggplot2 package [89]. Transcription levels are log_2_ read counts per kilobase of exon per million library reads (RPKM). For trace graphs as shown in S7–S13 Figs transcript coverage was calculated as explained for the ChIP-seq experiments to obtain counts per million reads (CPM). SM clusters are annotated as described by Inglis and coworkers [72]. All data are available at NCBI GEO under the accession number GSE72126.

### Chromatin immunoprecipitation (ChIP) and high- throughput sequencing (ChIP-seq)

Chromatin immunoprecipitation was performed as described in [18] Chromatin was incubated with antibodies specific to H3K4me3 (Abcam, 8580), H3K9me3 (Active Motif, 39161), H3K36me3 (Abcam, 9050), H3Ac (Millipore, 06–599) or Histone H3 C-terminus (Abcam, 1791) and Dynabeads Protein A (Invitrogen). Precipitated DNA from two biological and two technical replicates was quantified by real-time PCR according to protocol (Bio-Rad) using iQ SYBR Green Supermix and normalized to input DNA or sequenced. Primers used in quantitative PCR were HPLC purified and are shown in S2 Table. For Illumina sequencing, ChIP-seq libraries were prepared using 10 ng of immunoprecipitated DNA following the instructions supplied with Illumina Tru-seq ChIP-seq kits (Illumina Cat# FC-121-2002). Illumina sequencing was performed using an Illumina NextSeq500 Instrument at the University of Georgia Genomics Facility. Short reads were mapped using Novoalign (NovoCraft) to the current Aspergillus genome annotation, obtained from the Aspergillus Genome Database [90]. Read numbers were counted for 10 base pair bins using sam tools and R, and the read density was normalized for total read number and visualized using the Integrated Genome Viewer or Integrated Genome Browser [91–93]. In detail: Metaplots (Figs 3A, 5A, 5B and S4–S6) were calculated from bam files using bedtools genomecov [94]. The sequencing coverage per base pair (bp) was calculated for the whole genome, normalized using a scaling factor (1000000/total mapped sequence read counts) that accounts for the different counts of mapped reads per sample to obtain counts per million mapped reads (CPM) to allow comparison between samples represented as trace files in sgr format. Using R scripts the sgr files were smoothed by averaging a window of 100 bp length that was slided by 10bp, thereby reducing computation demand (10bp binning). Gene start/stop codon position, length and strand were retrieved from gff file provided by Aspergillus Genome Database [90] and 2kb of each gene (500bp upstream + 1500bp downstream of ATG in case of H3Ac and H3K4me3 or 1500bp upstream + 500bp downstream of stop codon for H3K36me) taken and averaged for the specified group (e.g. transcription strength). Detailed R scripts can be obtained from the authors.

Per gene levels of ChIP-seq data were calculated as transcript sequences counting reads per gene and were normalized to the ORF length (in comparison to the exon length) to obtain reads per million reads per kb ORF length (RPKM).All data are available at NCBI GEO under the accession number GSE72126.

### Analysis of natural product biosynthesis by HPLC-MS/MS

Small pieces of *kdmBΔ* and the wildtype strain, grown on YMG agar, were used to inoculate 100 mL of AMM and ZM_1/2_ medium in 500 mL Erlenmeyer flasks. The flasks were kept on a rotary shaker at 37°C and 160 rpm until the glucose was consumed (64 hours for AMM and 9 days for ZM_1/2_). pH value and glucose content of the culture fluid were monitored as described previously [95]. After harvesting, mycelium and culture fluid were separated by filtration. The culture fluid was extracted with the same volume of ethyl acetate twice, the combined organic layers were dried over sodium sulfate and the solvent was evaporated in vacuo (40°C) to provide the crude extract. The wet mycelium was extracted with 100 ml of acetone for 30 min in an ultrasonic bath (25°C) and the organic solvent was evaporated in vacuo (40°C). The remaining aqueous residue was diluted with 20 ml of H_2_O and extracted with the same volume of ethyl acetate twice. After drying over sodium sulfate, the organic solvent was removed in vacuo (40°C) to yield the crude mycelial extract. The extracts were dissolved in methanol, filtered through SPE C18 cartridges and subjected to mass spectrometric analyses.

All analyses were performed on Agilent 1260 Infinity Systems with diode array detector and C18 Acquity UPLC BEH column (2.1 × 50 mm, 1.7 μm) from Waters. Solvent A: H_2_O + 0.1% formic acid, solvent B: AcCN + 0.1% formic acid, gradient system: 5% B for 0.5 min increasing to 100% B in 19.5 min, maintaining 100% B for 5 min, flowrate = 0.6 mL min^−1^, UV detection 200–600 nm. LC-MS and MS/MS spectra were recorded on an ion trap MS (amaZon speed, Bruker) with an electrospray ionization source. Experiments were performed using positive and negative ionization modes. The capillary voltage of the ion source was 4500V and the nebulizer gas was set to 4 bar with drying gas flow of 12 L/min. For MS^2^ experiments SmartFrag was used with CID voltage of 1V and amplitude ramping of 60–180% (fragmentation time 40 ms, Cutoff 17%). HR-MS spectra were recorded on a time-of-flight (TOF) MS (MaXis, Bruker) with electrospray ionization source using positive ionization mode.

For data analysis and calculation of molecular formulas, including the isotopic pattern, dataAnalysis (Version 4.2) from Bruker was used. Compound search was performed using Dictionary of Natural Products (CRC Press) and Antibase 2010 (Wiley-VCH). ESI-MS, ESI-MS^2^ and UV-Vis absorption spectra of identified metabolites were compared to corresponding literature data [96–104].

## Supporting Information

S1 Fig KdmB has demethylase activity but low specificity *in vitro*. **A**. Schematic representation of the KdmB-GST fusion heterologously expressed in *E*. *coli*. The Coomassie stained SDS-PAGE Gel shows the purified GST-KdmB fusion protein and the arrow indicates protein migration at the expected size of around 130 kDa. 1–3 μg of purified GST-KdmB were loaded onto 10% SDS-PAGE gel. Lines 1–5 show different elution fractions. **B.** Western blot with the products of the *in vitro* demethylase assays. (+) Calf thymus histones were incubated with cofactors (α-ketoglutarate and Fe^2+^) and GST-purified KdmB. (-) Control reaction without the cofactors. 1 μg of calf thymus histones from both reactions (+) and (-) were loaded on 15% SDS-PAGE gel and the indicated HPTMs were analysed with the corresponding primary antibodies. A single replicate experiment was probed with H3 acetyl antibodies C. Densitometric quantification of Western blot signals from demethylase reactions (DeMt) (+) and the control reaction without the cofactors (-). A total of three DeMt reactions are plotted for each methyl antibody. Results are normalized to the histone H3 C- term signal which was arbitrarily set to a value of 1 (See Materials and Methods).
(TIF)

S2 Fig LC- MS/MS Base Peak Chromatograms (BPC). Ratios of differently modified variants of the histone H3 K_9_STGGK_14_APR and H3 K_27_AAPSTGGVK_36_K_37_PHR peptide from WT and *kdmBΔ*.
(TIF)

S3 Fig *kdmB* deletion does not alter the growth rate. (A) Measurement of glucose and nitrate concentration in *Aspergillus* Minimal Medium (AMM) supernatants from WT and *kdmB*Δ 22h cultures. (B) Equal amount of WT and *kdmB*Δ spores were point inoculated on solid AMM. Picture was taken after 48h.
(TIF)

S4 Fig Comparison between wild type and *kdmB*Δ cells for their association between H3K4me3 levels and gene transcription in 48 h SM cultures. Metaplots for H3K4me3 levels in genes belonging to the group with low average H3K4me3 levels in the wild type (log_2_ RPKM ≤ 5; group 5 and group 6 genes) and high average H3K4me3 levels in the wild type (log_2_ RPKM > 5; group 7 and group 8 genes). Similar to the analysis performed for cells grown under PM conditions (Fig 5), these respective groups were analyzed for their transcriptional behavior in the *kdmB* mutant under SM conditions. Genes with higher transcription in the wild type (WT) where KdmB acts as activator are shown in the bar graph with positive values (group 5 and group 7genes) whereas a negative value is assigned to genes which are stronger expressed in the mutant and thus repressed by KdmB activity (group 6 and group 8 genes, *kdmB*Δ).
(TIF)

S5 Fig Acetylation levels of the genes de-regulated in the *kdmB*Δ mutant largely mirror transcriptional activities. The genes deregulated in the *kdmB* mutant and previously subdivided into four differentially methylated H3K4me3 groups (group 1 to group 4 genes, Fig 5) were subjected here to analysis of acetylation levels. Identical to the bioinformatic procedure applied for H3K4me3 analysis, the global comparison of H3 acetylation levels was performed in the region starting 500 bp upstream from ATG up to 1500 bp downstream of genes differentially regulated between WT and *kdmBΔ* during primary metabolism. Levels of H3ac counts ([CPM]Ac) at each base pair (bp) in these genes are shown as line graphs in wild type (red lines) or in the *kdmB* mutant (green lines). The level and direction of differential expression for each deregulated gene in the group is shown in the middle panels for A and B (see Fig 5). RPKM, Reads per kb of ORF per million library reads.
(TIF)

S6 Fig KdmB does not influence chromosomal distribution of H3K36me3. **(A) H3K36me3 pattern of genes in primary metabolism.** The graph shows a comparison of H3K36me3 levels between WT and *kdmBΔ*. Because H3K36 methylation is located predominantly at the last third of reading frames, the predicted STOP codons of all genes were aligned and set as starting point of the analysis. Thus, H3K36me3 levels are represented from stop codons until 1500 bp upstream of the stop into the body of the reading frame. Analysis is based on cells in the primary metabolic phase (nutrient sufficiency). Similarly to H3K4me3 analysis, H3K36me3 signals were arbitrarily divided into two categories. (L): no/ low H3K36me3 [log_2_ (RPKM) ≤2] or (H): high H3K36me3 [log_2_ (RPKM) >2]. Red lines indicate WT, green lines indicate *kdmBΔ*. **(B) Differential gene expression analysis in the L or H H3K36me3 groups.** The bar blots depict genes within these two categories which are differentially transcribed between the WT and *kdmBΔ*, p<0.01. In the no/low RPKM category (left panel) only 112 genes need KdmB as activator whereas at the high K36me3 group 644 genes depend on KdmB activation (directly or indirectly for both groups). Similarly, 44 genes and 592 genes are under KdmB repression in the low and in the high H3K36me3 group, respectively.
(TIF)

S7 Fig Genome viewer image of the chromatin landscape and transcription profile of the penicillin gene cluster during primary (PM) and secondary metabolism (SM) in WT cells. The penicillin (PEN) cluster (red box, as defined in [105]) is located between heterochromatic domains at the subtelomeric region of chromosome VI. The cluster is known to be partially active already under primary metabolic conditions [106]. The colour code of the tracks represents the different modifications which are depicted next to the figure and described in detail the text. %GC, relative GC content of the analysed region. CPM, transcript counts per million of mapped reads in the RNA-seq analysis representing transcriptional activity of the locus. A control ChIP (No Ab) without any specific HPTM antibody added to the sample was performed to detect non-specific ChIP-seq signals (blue track).
(EPS)

S8 Fig Distribution of the analysed histone marks [log_2_CPM] and RNA-seq reads (log_2_ RPKM) in and around the sterigmatocystin (ST) gene cluster during primary (17 h) and secondary (48 h) metabolism in the WT. Structural genes belonging to the biosynthetic gene cluster are indicated by the red boxed area. The genes belonging to the cluster have been previously defined [75] and continuously named from *stcA* to *stcW*. The numbers below the tracks indicate the position of the cluster which extends for around 60 kbp on chromosome IV.
(EPS)

S9 Fig Distribution of the analysed histone marks [log_2_CPM] and RNA-seq reads (log_2_ RPKM) in and around the austinol (AUS) gene cluster during primary (17 h) and secondary (48 h) metabolism in the WT. Structural genes belonging to the two split biosynthetic gene cluster responsible for the biosynthesis of austinol are indicated by the two red boxed areas. The genes belonging to the biosynthetic pathway have been previously defined [73, 107] and named from *ausA* to *ausN*. The numbers below the tracks indicate the position of the two split clusters on chromosome VIII.
(EPS)

S10 Fig Distribution of selected histone marks [log_2_CPM] and RNA-seq reads (log_2_ RPKM) of the neighboring orsellinic acid (ORS) gene cluster [103] and DBA gene cluster [108]. Distribution of the analysed histone marks [log_2_CPM] and RNA-seq reads (log_2_ RPKM) in and around the clusters are shown for wild type cells in primary (17 h) and secondary (48 h) metabolism phase. Structural genes belonging to the biosynthetic gene cluster are indicated by the red boxed areas corresponding to previously defined genes. The genes belonging to the cluster have been previously defined. The numbers below the tracks indicate the position of the two biosynthetic clusters on chromosome II.
(EPS)

S11 Fig Distribution of selected histone marks [log_2_CPM] and RNA-seq reads (log_2_ RPKM) of the terrequinone (TDI) gene cluster. Distribution of the analysed histone marks [log_2_CPM] and RNA-seq reads (log_2_ RPKM) in and around the cluster are shown for wild type cells in primary (17 h) and secondary (48 h) metabolism phase. Structural genes belonging to the biosynthetic gene cluster are indicated by the red boxed areas corresponding to previously defined genes. The genes belonging to the cluster have been previously defined [109]. The numbers below the tracks indicate the position of the two biosynthetic clusters on chromosome V.
(EPS)

S12 Fig Distribution of selected histone marks [log_2_CPM] and RNA-seq reads (log_2_ RPKM) of the asperthecin (APT) gene cluster. Distribution of the analysed histone marks [log_2_CPM] and RNA-seq reads (log_2_ RPKM) in and around the cluster are shown for wild type cells in primary (17 h) and secondary (48 h) metabolism phase. Structural genes belonging to the biosynthetic gene cluster are indicated by the red boxed areas corresponding to previously defined genes [73]. The numbers below the tracks indicate the position of the two biosynthetic clusters on chromosome I.
(EPS)

S13 Fig Distribution of selected histone marks [log_2_CPM] and RNA-seq reads (log_2_ RPKM) of the monodictiphenon (MDP), gene Distribution of selected histone marks [log_2_CPM] and RNA-seq reads (log_2_ RPKM) of the asperthecin (APT) gene cluster. Distribution of the analysed histone marks [log_2_CPM] and RNA-seq reads (log_2_ RPKM) in and around the cluster are shown for wild type cells in primary (17 h) and secondary (48 h) metabolism phase. Structural genes belonging to the biosynthetic gene cluster are indicated by the red boxed areas corresponding to previously defined genes [98]. The numbers below the tracks indicate the position of the two biosynthetic clusters on chromosome VIII.
(EPS)

S1 Table List of strains used in this study. (DOCX)

S2 Table List of oligonucleotides used in this study. (DOCX)

S3 Table LC-MS and LC-MS2 data of detected metabolites (a) HR-ESI-MS; b) LC/ESI-MS; c) LC/ESI+-MS2. (TIF)
