# Supplementary figures and images for: KdmB, a Jumonji Histone H3 Demethylase, Regulates Genome-Wide H3K4 Trimethylation and Is Required for Normal Induction of Secondary Metabolism in Aspergillus nidulans
Source: PLoS Genet. 2016 Aug 22;12(8):e1006222. doi: 10.1371/journal.pgen.1006222 (PMC4993369; doi:10.1371/journal.pgen.1006222)

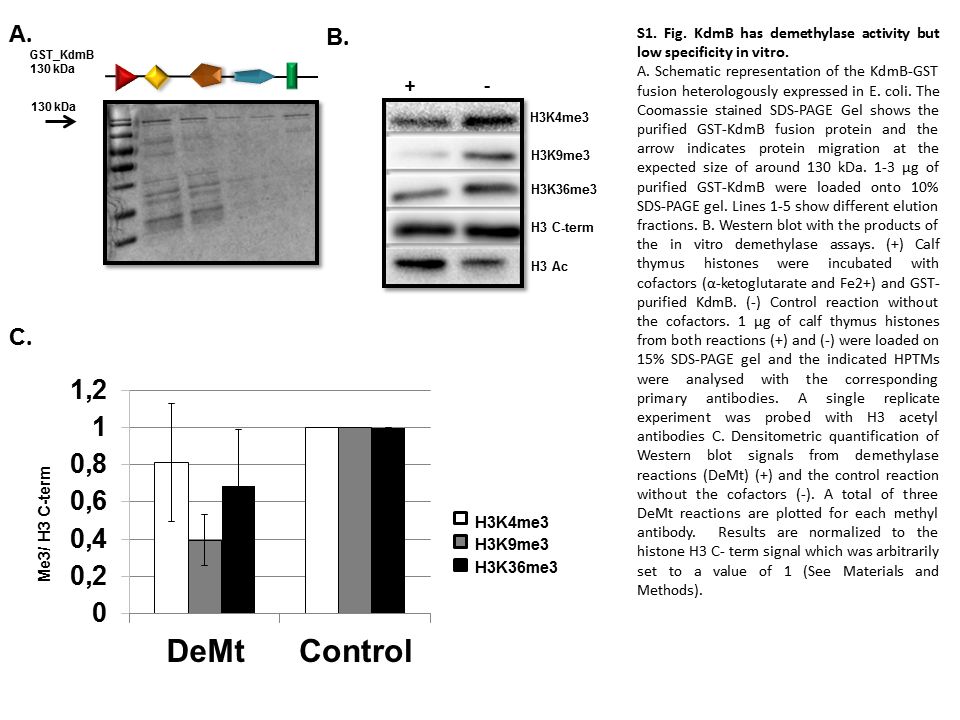

Supplement: S1 Fig — A. Schematic representation of the KdmB-GST fusion heterologously expressed in E. coli. The Coomassie stained SDS-PAGE Gel shows the purified GST-KdmB fusion protein and the arrow indicates protein migration at the expected size of around 130 kDa. 1–3 μg of purified GST-KdmB were loaded onto 10% SDS-PAGE gel. Lines 1–5 show different elution fractions. B. Western blot with the products of the in vitro demethylase assays. (+) Calf thymus histones were incubated with cofactors (α-ketoglutarate and Fe2+) and GST-purified KdmB. (-) Control reaction without the cofactors. 1 μg of calf thymus histones from both reactions (+) and (-) were loaded on 15% SDS-PAGE gel and the indicated HPTMs were analysed with the corresponding primary antibodies. A single replicate experiment was probed with H3 acetyl antibodies C. Densitometric quantification of Western blot signals from demethylase reactions (DeMt) (+) and the control reaction without the cofactors (-). A total of three DeMt reactions are plotted for each methyl antibody. Results are normalized to the histone H3 C- term signal which was arbitrarily set to a value of 1 (See Materials and Methods). (TIF) [file pgen.1006222.s001.tif]

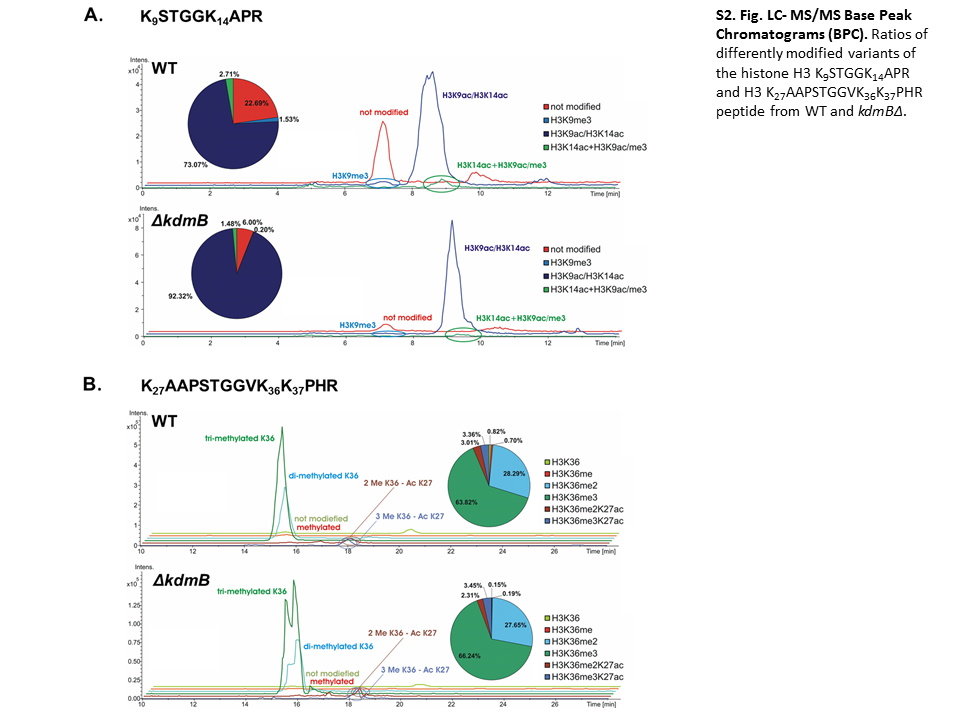

Supplement: S2 Fig — Ratios of differently modified variants of the histone H3 K9STGGK14APR and H3 K27AAPSTGGVK36K37PHR peptide from WT and kdmBΔ. (TIF) [file pgen.1006222.s002.tif]

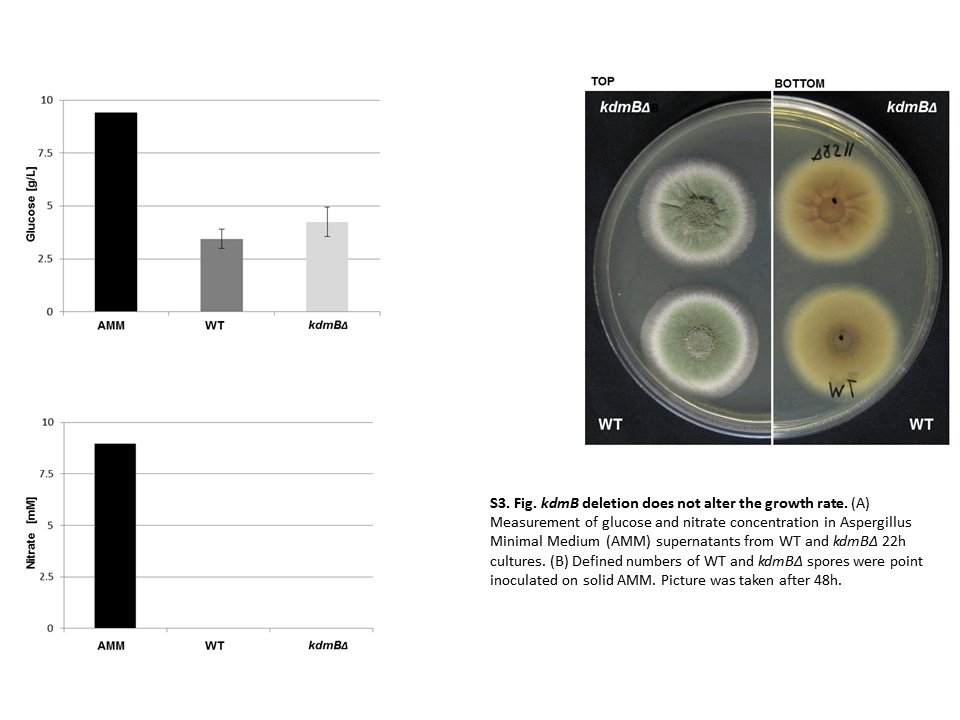

Supplement: S3 Fig — (A) Measurement of glucose and nitrate concentration in Aspergillus Minimal Medium (AMM) supernatants from WT and kdmBΔ 22h cultures. (B) Equal amount of WT and kdmBΔ spores were point inoculated on solid AMM. Picture was taken after 48h. (TIF) [file pgen.1006222.s003.tif]

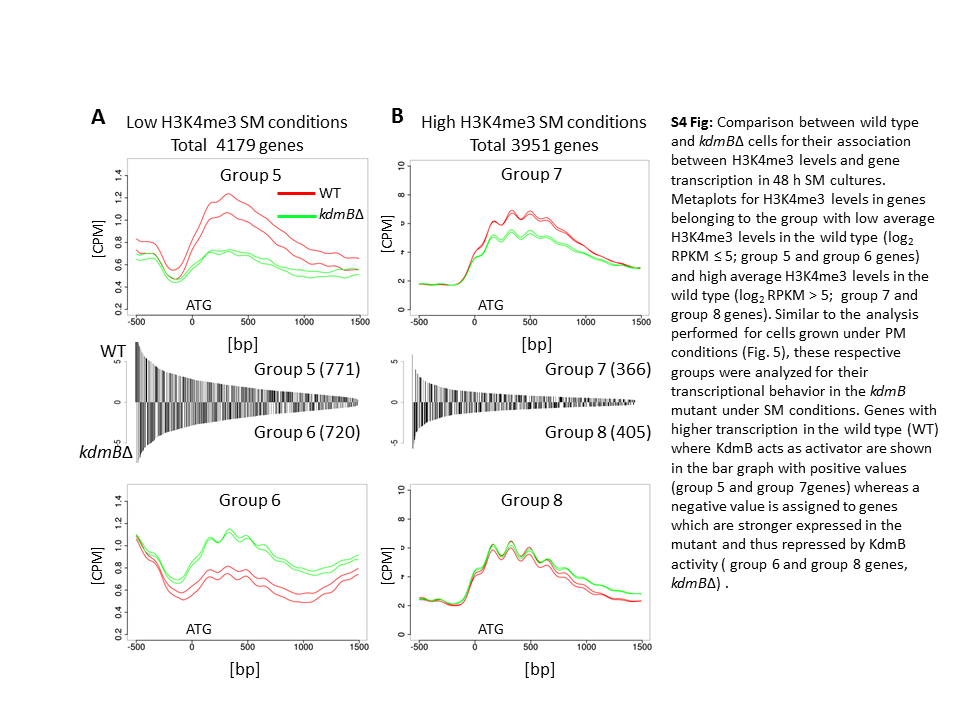

Supplement: S4 Fig — Metaplots for H3K4me3 levels in genes belonging to the group with low average H3K4me3 levels in the wild type (log2 RPKM ≤ 5; group 5 and group 6 genes) and high average H3K4me3 levels in the wild type (log2 RPKM > 5; group 7 and group 8 genes). Similar to the analysis performed for cells grown under PM conditions (Fig 5), these respective groups were analyzed for their transcriptional behavior in the kdmB mutant under SM conditions. Genes with higher transcription in the wild type (WT) where KdmB acts as activator are shown in the bar graph with positive values (group 5 and group 7genes) whereas a negative value is assigned to genes which are stronger expressed in the mutant and thus repressed by KdmB activity (group 6 and group 8 genes, kdmBΔ). (TIF) [file pgen.1006222.s004.tif]

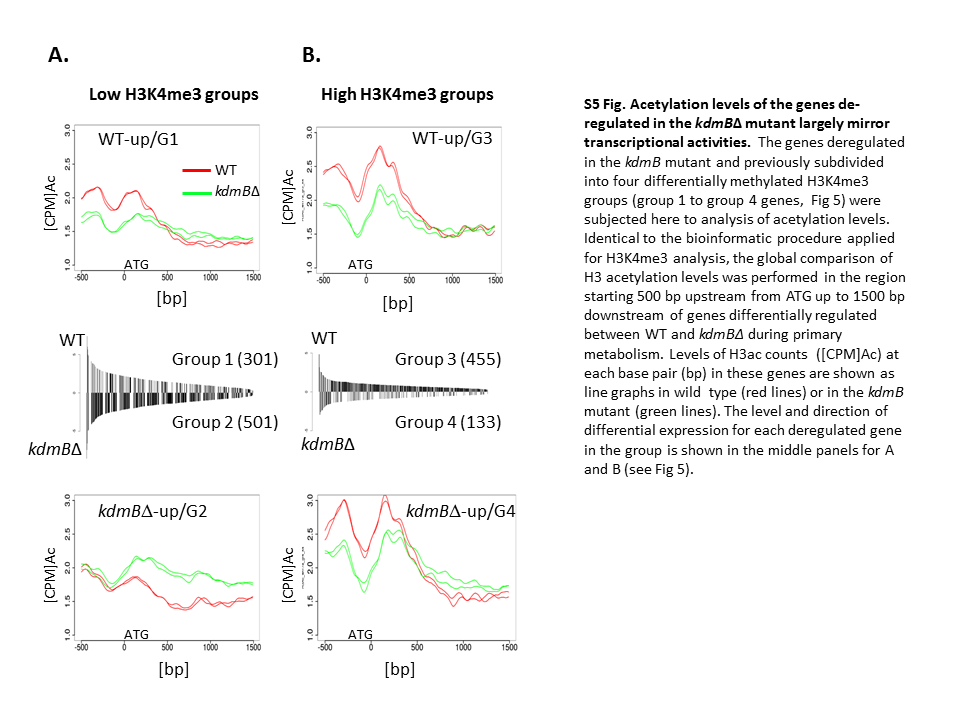

Supplement: S5 Fig — The genes deregulated in the kdmB mutant and previously subdivided into four differentially methylated H3K4me3 groups (group 1 to group 4 genes, Fig 5) were subjected here to analysis of acetylation levels. Identical to the bioinformatic procedure applied for H3K4me3 analysis, the global comparison of H3 acetylation levels was performed in the region starting 500 bp upstream from ATG up to 1500 bp downstream of genes differentially regulated between WT and kdmBΔ during primary metabolism. Levels of H3ac counts ([CPM]Ac) at each base pair (bp) in these genes are shown as line graphs in wild type (red lines) or in the kdmB mutant (green lines). The level and direction of differential expression for each deregulated gene in the group is shown in the middle panels for A and B (see Fig 5). RPKM, Reads per kb of ORF per million library reads. (TIF) [file pgen.1006222.s005.tif]

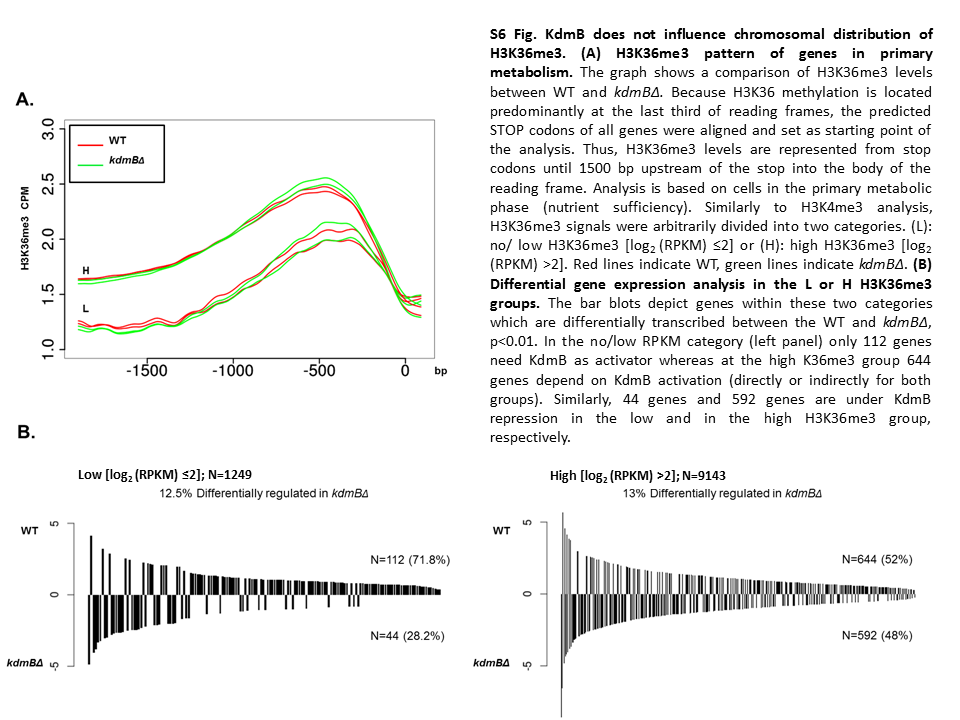

Supplement: S6 Fig — (A) H3K36me3 pattern of genes in primary metabolism. The graph shows a comparison of H3K36me3 levels between WT and kdmBΔ. Because H3K36 methylation is located predominantly at the last third of reading frames, the predicted STOP codons of all genes were aligned and set as starting point of the analysis. Thus, H3K36me3 levels are represented from stop codons until 1500 bp upstream of the stop into the body of the reading frame. Analysis is based on cells in the primary metabolic phase (nutrient sufficiency). Similarly to H3K4me3 analysis, H3K36me3 signals were arbitrarily divided into two categories. (L): no/ low H3K36me3 [log2 (RPKM) ≤2] or (H): high H3K36me3 [log2 (RPKM) >2]. Red lines indicate WT, green lines indicate kdmBΔ. (B) Differential gene expression analysis in the L or H H3K36me3 groups. The bar blots depict genes within these two categories which are differentially transcribed between the WT and kdmBΔ, p<0.01. In the no/low RPKM category (left panel) only 112 genes need KdmB as activator whereas at the high K36me3 group 644 genes depend on KdmB activation (directly or indirectly for both groups). Similarly, 44 genes and 592 genes are under KdmB repression in the low and in the high H3K36me3 group, respectively. (TIF) [file pgen.1006222.s006.tif]

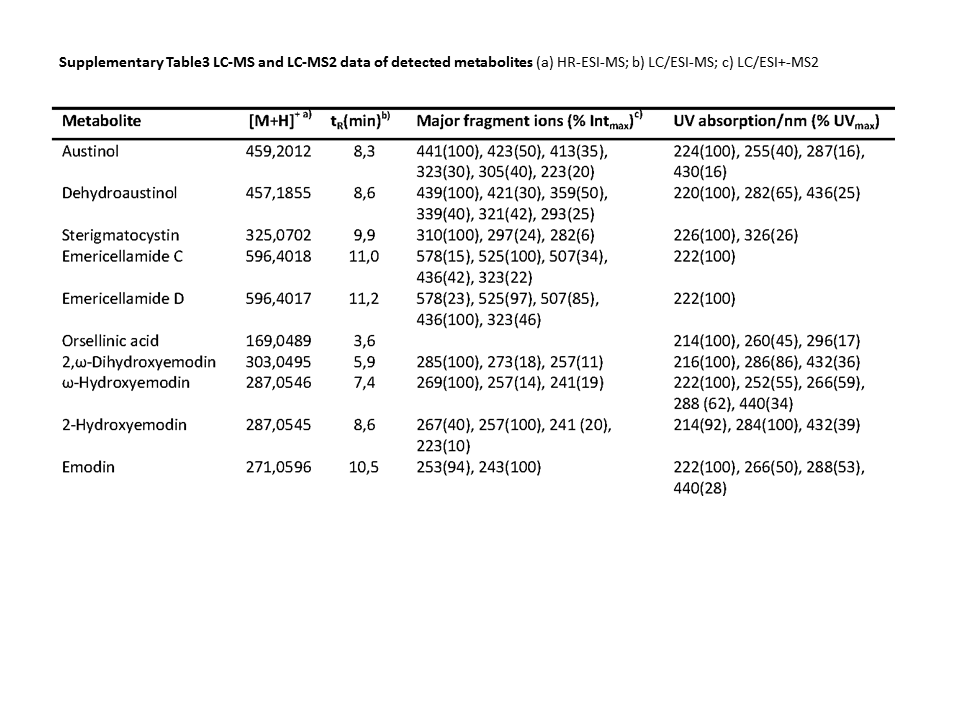

Supplement: S3 Table — (TIF) [file pgen.1006222.s016.tif]
