# Supplementary material for: KdmB, a Jumonji Histone H3 Demethylase, Regulates Genome-Wide H3K4 Trimethylation and Is Required for Normal Induction of Secondary Metabolism in Aspergillus nidulans
Source: PLoS Genet. 2016 Aug 22;12(8):e1006222. doi: 10.1371/journal.pgen.1006222 (PMC4993369; doi:10.1371/journal.pgen.1006222)
Supplement: S1 Table — (DOCX) [file pgen.1006222.s014.docx]

**Supplementary Table 1: List of strains used in this study**

*A.nidulans* strains: Reference

**Ag2 *kdmBΔ*** *kdmB Δ::Af_ribo, nkuA Δ::argB2, pabaB22, pyrG89, veA1* this study

**Ag5 *kdmBΔ*** *kdmB Δ::Af_ribo, nkuA Δ::argB2, pabaB22, veA1* this study

**WT** *pabaA1 veA1* Pontecorvo et al. 1953

*E.coli* strains:

**Rosetta™kdmB_jmjC** *pGEX_4T1_KdmB_jmjC* F^-^ *ompT hsdS*_B_(r_B_^-^ m_B_^-^)

*gal dcm* (DE3) pLysSRARE (Cam^R^) (Amp^R^) this study

1. Pontecorvo G, Roper JA, Hemmons LM, Macdonald KD, Bufton AW. The genetics of Aspergillus nidulans. Adv Genet. 1953;5:141-238. PubMed PMID: 13040135.
