# Supplementary material for: KdmB, a Jumonji Histone H3 Demethylase, Regulates Genome-Wide H3K4 Trimethylation and Is Required for Normal Induction of Secondary Metabolism in Aspergillus nidulans
Source: PLoS Genet. 2016 Aug 22;12(8):e1006222. doi: 10.1371/journal.pgen.1006222 (PMC4993369; doi:10.1371/journal.pgen.1006222)
Supplement: S2 Table — (DOCX) [file pgen.1006222.s015.docx]

**Supplementary Table 2: List of oligonucleotides used in this study**

a)quantitative PCR

aflR_q _F AGCCCAGCTGGTGCTGAGCGAGCTATAC Reyes-Dominguez et al. 2010

aflR_q_R CCAGGGTGGTCGACGACAAGGGGGT Reyes-Dominguez et al. 2010

stcO_q_F CCCGTGCTTGCGGTTCAAGT this study

stcO_q_R GCACGGCTGGCAATCTCACA this study

actA_q_F CACCGGTATCGTCCTTGACT Reyes-Dominguez et al. 2010

actA_q_R CTCAGCGGTAGTGGAGAAGG Reyes-Dominguez et al. 2010

benA_q_F GATGGCTGCCTCTGACTTCCG this study

benA_q_R GCGCATCTGGTCCTCAACCTC this study

tdiB_q_F CCCTTCGACCCCAGCATCAA this study

tdiB_q_R GCGGGGGTCAGGTAGAGAGCA this study

ipnA_q_F GGAGACGACCAAGCAGCCAAA Gacek-Matthews et al. 2015

ipnA_q_R TTTTCCCGGGGATGGACAGG Gacek-Matthews et al. 2015

orsB_q_F GGGCGACTCGAGGCAATGAA Nützmann et al. 2011

orsB_q_R GACTGCGAAGGCAGCGAAGC Nützmann et al. 2011

acvA_q_F CAATGGCGACGTTGATGCAG this study

acvA_q_R ATGCCGGCTTTCCAACCTTC this study

b) ChIP

aflR_p_F AACCCTTGAAACCCATAGCCAGTAAA Reyes-Dominguez et al. 2010

aflR_p_R GATATTTGCATATGATACAGGCCC Reyes-Dominguez et al. 2010

benA_p_F GATGGCTGCCTCTGACTTCCG Gacek-Matthews et al. 2015

benA_p_R GCGCATCTGGTCCTCAACCTC Gacek-Matthews et al . 2015

orsB_p_F GCTCCAGCTGCTGTGCCCTA Nützmann et al. 2011

orsB_p_R TAAGGCAGACCGGCCATTCA Nützmann et al. 2011

ipnA_p_F TGGTCGGCTCGATGTCCAAG this study

ipnA_p_R GGGCAAGCAGTTGAGACTGATGAA this study

stcO_p_F TGGCAGGCAGCGTTTATGGA this study

stcO_p_ R AGAGAAGGTGCAGGAATTATGCAGGTA this study

c) *kdmB* heterologous expression

8211jmjC_R_Xhol TGATTCTCGAGTTACAACTGGGGCTTCGGATCATC this study

pGEX_8211_Xmal_F GGATCCCCGGAATTCCCGGGAATGGTGGCTCCGGCTGCAATGG this study

kdmB_R TTAAGCGGCCGCAGTGGC this study

kdmB_R_1800 GTCCGCACCGTATTCAACTT this study

kdmB_R_3101 AGGCGGAATTTCTCGATCTC this study

kdmB_R_4800 GAGATGAGGGGAGACCATGA this study

d) *kdmB* knock out

AN8211_UPf TCCAACTCATATCAAGATGGTCGC this study

AN8211_UP_NESTEDF TAAGGAGCAGTATGTAGATAATGAC this study

AN8211_UPR GTCTTGGGTGCGATAGCTGGACAATTAGG this study

AN8211_riboFum_F this study GCGCGCACCCCGTTCGATCCTAATTGTCCAGCTATCGCACCCAAGACGATCGCGGAGCGCAACGGGCAGCTTG

AN8211_riboFum_R this study

GTAGAATAGTGGTGAGGAATAAGATGTTGAATGAGGCAGATGGAATGGATTGGTTCTGGCTCAACCAGCCCCG

AN8211_downF TTCCATCTGCCTCATTCAACATCTTATTCC this study

AN8211_downR ATGGAGTATAGTGACTAACGCACAC this study

AN8211_down_nested_R CTCAGCCTCAGCATCAACAACACAC this study

AN8211_orfF AATCCCACCCCTCTAGAGGCTG this study

AN8211_orfR CCTGGAATCCCATACCAAGTCTTG this study
